# Genome reorganization of the *GmSHMT* gene family in soybean showed a lack of functional redundancy in resistance to soybean cyst nematode

**DOI:** 10.1038/s41598-018-37815-w

**Published:** 2019-02-06

**Authors:** Naoufal Lakhssassi, Gunvant Patil, Sarbottam Piya, Zhou Zhou, Azam Baharlouei, My Abdelmajid Kassem, David A. Lightfoot, Tarek Hewezi, Abdelali Barakat, Henry T. Nguyen, Khalid Meksem

**Affiliations:** 1 Department of Plant, Soil and Agricultural Systems, Southern Illinois University, Carbondale, IL 62901 USA; 2 Division of Plant Sciences, University of Missouri, Columbia, MO 65201 USA; 3 Department of Agronomy and Plant Genetics, University of Minnesota, St. Paul, MN 55108 USA; 4 Department of Plant Sciences, University of Tennessee, Knoxville, TN 37996 USA; 5 Department of Biological Sciences, Fayetteville State University, Fayetteville, NC 28301 USA; 6 Department of Biology, University of South Dakota, Vermillion, SD 57069 USA

## Abstract

In soybeans, eighteen members constitute the serine hydroxymethyltransferase (*GmSHMT*) gene family, of which the cytosolic-targeted *GmSHMT08*c member has been reported to mediate resistance to soybean cyst nematode (SCN). This work presents a comprehensive study of the *SHMT* gene family members, including synteny, phylogeny, subcellular localizations, haplotypes, protein homology modeling, mutational, and expression analyses. Phylogenetic analysis showed that *SHMT* genes are divided into four classes reflecting their subcellular distribution (cytosol, nucleus, mitochondrion, and chloroplast). Subcellular localization of selected GmSHMT members supports their *in-silico* predictions and phylogenetic distribution. Expression and functional analyses showed that *GmSHMT* genes display many overlapping, but some divergent responses during SCN infection. Furthermore, mutational analysis reveals that all isolated EMS mutants that lose their resistance to SCN carry missense and nonsense mutations at the *GmSHMT08c*, but none of the *Gmshmt08c* mutants carried mutations in the other *GmSHMT* genes. Haplotype clustering analysis using the whole genome resequencing data from a collection of 106 diverse soybean germplams (15X) was performed to identify allelic variants and haplotypes within the *GmSHMT* gene family. Interestingly, only the cytosolic-localized GmSHMT08c presented SNP clusters that were associated with SCN resistance, supporting our mutational analysis. Although eight *GmSHMT* members respond to the nematode infestation, functional and mutational analysis has shown the absence of functional redundancy in resistance to SCN. Structural analysis and protein homology modeling showed the presence of spontaneous mutations at important residues within the GmSHMT proteins, suggesting the presence of altered enzyme activities based on substrate affinities. Due to the accumulation of mutations during the evolution of the soybean genome, the other GmSHMT members have undergone neofunctionalization and subfunctionalization events.

## Introduction

Soybean [*Glycine max* (L.) Merr.] is the most widely consumed legume crop worldwide. However, soybean production is limited by the presence of the soybean cyst nematode (SCN; *Heterodera glycines* I.), causing over $1 billion in yield losses annually in the U.S.^1^. Most of the SCN resistant soybean lines are mainly derived from two types; PI 88788 and Peking. Peking-type resistance to SCN requires resistant alleles at two loci, the *Rhg4* and the *rhg1-a*^2^. The two genes underlying resistance to SCN have been isolated, encoding the soluble NSF attachment protein (*GmSNAP18*) at the *rhg1-*a locus and the serine hydroxymethyltransferase (*GmSHMT08c*) at the *Rhg4* locus^3,4^.

The serine hydroxylmethyltransferase (*SHMT;* EC 2.1.2.1) gene family is present in all plant and animal lineages. SHMT is an ubiquitous, homotetrameric enzyme with a key role in one-carbon metabolism, methionine synthesis, and maintenance of redox homeostasis during photorespiration^5,6^. SHMT is involved in the catalysis of reversible hydroxymethyl group transfer and interconversion of serine/glycine and tetrahydrofolate (THF)/5,10-methyleneTHF via transaldimination reactions^7–9^. In humans, mutations in *SHMT* have been shown to be involved in multiple diseases including cancers and cardiovascular diseases^10–12^.

In plants, the *SHMT* gene family has been studied in the plant model *Arabidopsis thaliana*. All seven members were reported to be localized within four intracellular compartments, with two in the nucleus and two in the cytosol, two in the mitochondrion, and one in the chloroplast^13^. In eudicots including pea, potato, spinach, and *A*. *thaliana*, SHMT activity was detected in most cell compartments including cytosol, mitochondria, nucleus, and plastids^14–17^. However, in monocots, only *Hordeum vulgare* has reported the presence of plastid-targeted SHMT enzyme activity^13,14^, but no orthologous gene could be found, inferring an aneupleurotic pathway or enzyme. In soybean, a member of the dicot gene family (*GmSHMT08c)* underlying the *Rhg4* loci has been reported to be involved in SCN resistance. It has been suggested that the SCN-resistant *Rhg4* allele emerged via artificial selection during the soybean domestication process^18^. However, little is known about the role of the other *GmSHMT* genes in plant abiotic stresses, and if the rest of the *GmSHMT* gene family (or some members) can play similar roles as the GmSHMT08 and may present functional redundancy or additive effect in resistance to SCN, as it has been reported recently in case of the *GmSNAP* gene family^19^. Analyzing the function of SHMT genes or any other gene family in soybean is a difficult task, since about 80% percent of soybean genes are duplicates^20–22^. Soybean breeding and targeting essential genes to improve important agronomic traits (i.e. oil, protein, yield, resistant to biotic and abiotic stresses) is challenging due to those duplication events and the presence of multiple gene copies^23,24^. The soybean genome encodes multiple chloroplastic, mitochondrial, nuclear, and cytosolic-localized GmSHMT classes. Mutations at the cytosol-targeted *GmSHMT08c* but not on the other *GmSHMT* members result in the loss of resistance to SCN in the resistant *c*.*v* Forrest lines^3^. Here, we report a detailed characterization of the *GmSHMT* gene family in soybean including structure, synteny, phylogeny, expression, homology modeling, subcellular localization, and mutational analyses.

## Results

### Duplication of *GmSHMT* in the soybean genome

Genome-wide analysis showed that the *GmSHMT* gene family in soybean is composed of at least twelve members named as *GmSHMT02*, *GmSHMT04*, *GmSHMT05*, *GmSHMT06*, *GmSHMT08* (with three genes on chromosome 08), *GmSHMT09*, *GmSHMT12*, *GmSHMT13*, *GmSHMT14*, and *GmSHMT18* (the number indicated the chromosome locations), in addition to a thirteenth member (*GmSHMT15*) corresponding to a truncated protein (Supplementary Fig. S1). Chromosome 08 carries three basal members of the *GmSHMT* gene family; *GmSHMT08c* (cytosolic), *GmSHMT08n* (nucleic), and *GmSHMT08m* (mitochondrial). Chromosome 13 carries a basal *GmSHMT13ch* (chloroplastic) and a multifunctional *GmSHMT13m* (mitochondrial). *GmSHMT* gene family members encode proteins that vary in size between 471aa and 603aa, except the *GmSHMT15* gene that encodes a truncated protein resulting in 244aa (Supplementary Fig. S1).

A number of genes with lower similarity to *SHMT* or associated with other protein domains were also found, increasing the number of *GmSHMT* gene family to eighteen members on the soybean genome (Supplementary Fig. S2). In fact, five additional GmSHMTs with multiple-bifunctional activities including a SHMT/threoninealdolase (Glyma.12g159200; GmSHMT12c, Glyma.15G090000; GmSHMT15c, and Glyma.16G108100; GmSHMT16c), a SHMT/wall-associated receptor kinase galacturonan-binding (Glyma.13G077700; GmSHMT13c), and a SHMT/bZIP transcription factor (Glyma.09G184300; GmSHMT09n) have been annotated in *Phytozome,* and their orthologs from the plant model were studied earlier (Supplementary Fig. S2)^25^. It has been reported that the SHMT/threoninealdolases and SHMT/wall-associated receptor kinase galacturonan-binding presented a cytosolic localization in yeast and Arabidopsis, respectively^26,27^. However, the Arabidopsis SHMT/bZIP transcription factor presented a nucleic localization^28^. Protein alignments showed that these *GmSHMTs* have diverged in their structure and function from the basal SHMT proteins. In this study, we studied only members of the *GmSHMT* family annotated as basal serine hydroxymethyltransferases.

To test the contribution of the soybean segmental duplications to the increase of soybean *SHMT* genes, we analyzed the distributions of *GmSHMT*s on chromosomal duplicated segments compiled from the Plant Genome Duplication Database^29–31^. Synteny analysis showed the presence of several commonly linked genes in duplicated blocks (Supplementary Fig. S3). Gene pairs *GmSHMT08c/GmSHMT05c*, *GmSHMT12n/GmSHMT08n*, *GmSHMT06n/GmSHMT04n*, *GmSHMT13ch/GmSHMT15ch*, and *GmSHMT13m/GmSHMT14m* were located on highly conserved duplicated blocks of ch08/ch05, ch12/ch08, ch06/ch04, ch13/ch15, in addition to an old duplication between ch13/ch14, respectively. These blocks encompassed 551, 14, 711, 391, and 24 conserved genes or anchors, respectively (Supplementary Table S1). Furthermore, *GmSHMT08m* was present in two different more diverged duplicated pairs (ch08/ch09, and ch08/ch02), with the presence of 24 and 10 additional conserved linked genes (Supplementary Fig. S3D). In addition, *GmSHMT02m* was found in two duplicated blocks located on ch02/14 (recent duplication) and ch02/18 (older duplication), with the presence of 248 and 7 additional conserved genes surrounding *GmSHMT02m*, respectively (Supplementary Fig. S3D; Supplementary Table S1).

### Phylogenetic analysis of the *SHMT* genes

**Figure 1:**
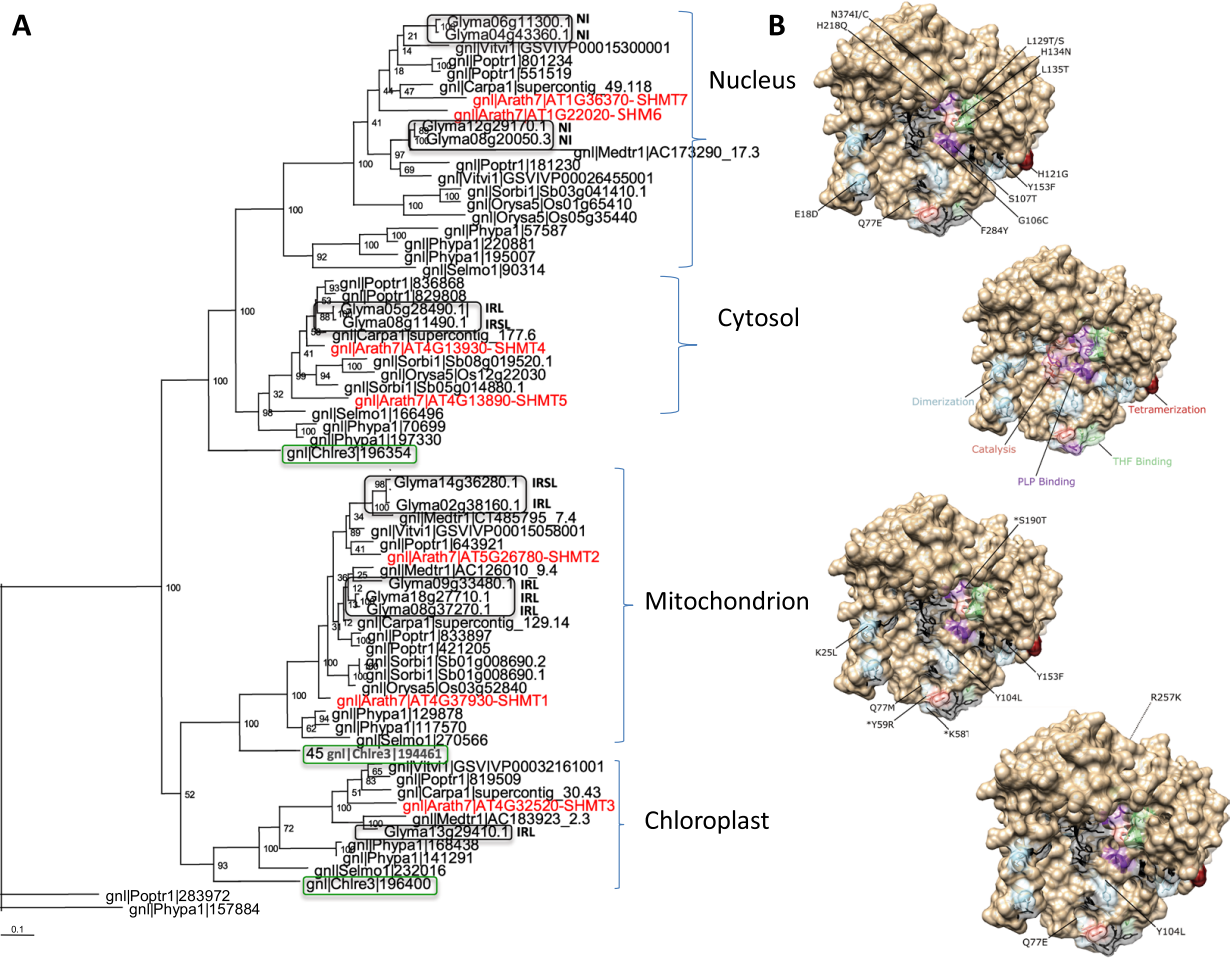
Phylogenetic tree of SHMT classes from sequenced plant species. (**A)** All SHMT proteins identified in five model plants; *C*. *reinhardtii* (algae; green box), *P*. *patens* (moss), *S*. *moellendorfii* (lycophyte), *O*. *sativa* (monocot), and *A*. *thaliana* (eudicot), in addition to *G*. *max* (soybean; black box) and other monocots and eudicots cytosolic, nucleic, chloroplastic, and mitochondrial-localized SHMTs were included in the analysis. SHMTs (in red) from *A*. *thaliana* belong to mitochondrial SHMT1 (AT4g37930) and SHMT2 (AT5g26781), the chloroplastic SHMT3 (AT4g32520), the cytosolic SHMT4 (AT4g13930) and SHMT5 (AT4g13890), in addition to the nucleic members SHMT6 (AT1g22020) and SHMT7 (AT1G36370). Glyma: *G*. *max*; Vitvi: *V*. *Vinifera*; Carpa: *C*. *papaya*; Arath: *A*. *thaliana*; Medtr: *M*. *truncatula*; Poptr: *P*. *trichocarpa*; Sorbi: *S*. *bicolor*; Orysa: *O*. *sativa*; Selmo: *S*. *moellendorfii*; *Phypa: P*. *patens*; Chlre: *C*. *reinhardtii*. **(B)** One SHMT subunit with highlighted catalytic sites, PLP and THF cofactor binding and oligomeric structural residues labelled. Domain variation analysis of the GmSHMT classes showing that most of the domain variation was observed within the nucleic-targeted GmSHMT class, with 14 domain variation out of 40, affecting protein structure (dimerization and tetramerization), substrate binding (including THF and PLP binding), and catalysis. **NI**: Transcripts Non-Induced under SCN infection; **IRL**: Transcripts Induced in Resistant line only under SCN infection; **IRSL**: Transcripts Induced in Resistant and Susceptible lines under SCN infection.

To elucidate the evolution of the *GmSHMT* gene family in soybean, phylogenetic analysis was conducted using genes from 22 sequenced plant species. The analysis separately grouped SHMTs into two groups and four classes corresponding to the cellular localization of these genes; nuclear, cytosol, mitochondria, and chloroplast-localized *GmSHMT*s. The two nuclear and cytosol-localized *GmSHMT*s classes clustered together, whereas the mitochondria and chloroplast-localized *GmSHMT*s classes formed a second cluster. Each class included sequences from various lineages: monocots, eudicots, mosses, lycophytes, and algae (Fig. 1). Surprisingly, the chloroplast-localized class did not include a monocot species. Extensive searches employing a large number of monocots including *O*. *sativa*, *Z*. *mays*, *S*. *bicolor*, *S*. *italica*, *H*. *vulgare*, *P*. *hallii*, *P*. *virgatum*, and *T*. *aestivum* failed to identify any *GmSHMT* member within the chloroplastic clade (Supplementary Fig. S4). The phylogenetic distribution of *SHMT* genes showed that several within-class duplicates are the result of duplication or polyploidization events at the species or lineage levels (Fig. 1).

### Structure analysis of intron-exons

Structural analysis revealed that organellar *GmSHMT*s had the highest number of exons and introns. As shown in Supplementary Fig. S5, all the five mitochondrial-localized *GmSHMTs* had 15 exons. The two chloroplastic genes have different number of exons; *GmSHMT13ch* had 11 exons while *GmSHMT15ch* presented 6 exons only and encodes for a truncated protein. Moreover, all four nuclear-localized GmSHMT members contained 4 exons. In contrast, the two cytosol-localized *GmSHMTs* had different numbers of exons, with *GmSHMT05c* and *GmSHMT08c* having 4 and 3 exons, respectively (Supplementary Fig. S5).

**Figure 2:**
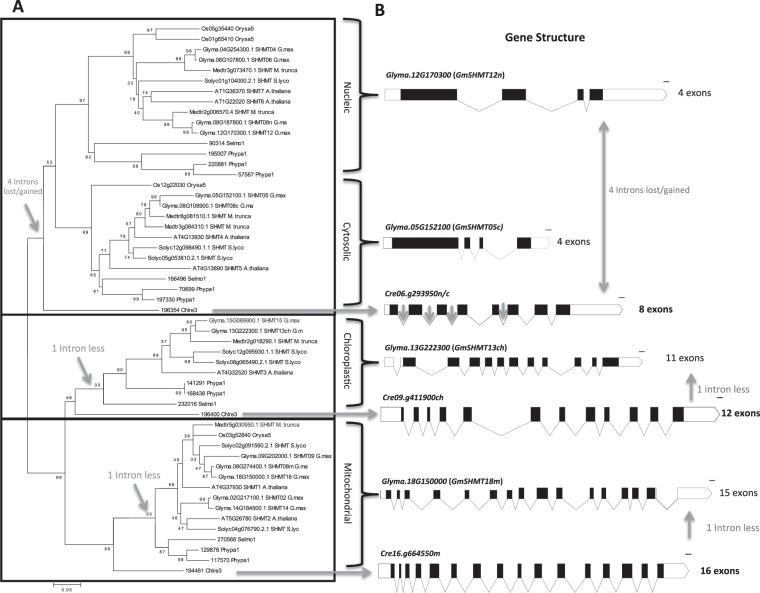
The evolution of *SHMT* genes. (**A)** SHMT gene divergence, duplication, and intron loss/gain events occurred during the transition from *C*. *reinhardtii*, representing a relative of the aquatic ancestor of all land plants, to the most ancestral land plants, and was maintained through all monocots and dicots. **(B)** *SHMT* gene structural analysis showed that an intron loss/gain event had occurred in the common ancestor of the nucleic/cytosolic-targeted *GmSHMTn/c*. The phylogenetic tree was generated using MEGA4 software package and the ClustalW algorithm, and calculated using the neighbor-joining method. The tree bootstrap values are indicated at the nodes (n = 1000). Gray arrows indicate the reported intron loss/gain events that occured during the transition from the relative of the aquatic ancestor of land plants. Gene structure of one *GmSHMT* representing each class is represented. The gene structure of the rest of the *GmSHMT* members can be found at Supplementary Fig. S5.

*Chlamydomonas reinhardtii* represents the polyphyletic chlorophytes, one of which was hypothesized to be a relative of the aquatic ancestor of all land plants, the green algae^32^. Comparison between *C*. *reinhardtii* and the land plant species showed that they present similar number of exons and introns (with land plants having 1 exon and 1 intron less) (Fig. 2). However, *Cre06*.*g293950 SHMT* gene from algae (clustering with both cytosolic and nucleic *SHMTs*) contained eight exons, while their cytosolic and nucleic counterparts have four exons only in both. This is maybe due to four intron loss/gain events that may have occurred in the ancestor of land plant (Fig. 2). For instance, either the algae or the land plant *SHMT* genes may have acquired or lost these introns during their lineage-specific evolution.

Moreover, the cytosolic *GmSHMT08c* presented different exon and intron structure between Forrest and WI82 soybean lines. The analysis shows evidence of alternative splicing variants and exon skipping events that may have occurred between the cytosolic *GmSHMT08c* from Forrest and *GmSHMT08c* from WI82 (Supplemental Fig. S5). The exon skipping event is common in plants and animals^33^.

### Subcellular localization of selected GmSHMTs

**Figure 3:**
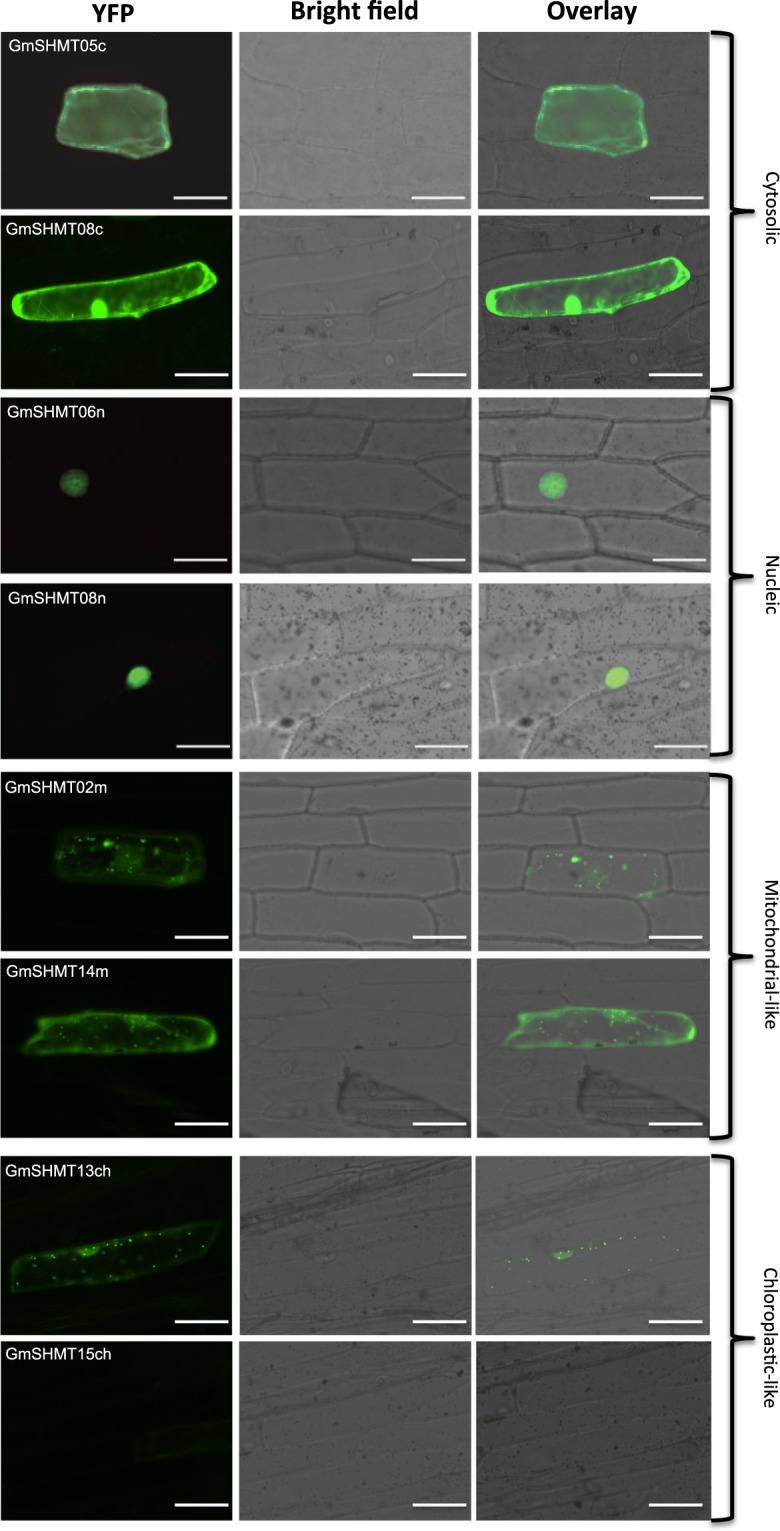
Subcellular localization of selected soybean *GmSHMT* genes belonging to the four GmSHMT classes. The coding sequences of the indicated *GmSHMT* genes were fused to the N-terminal end of the eYFP and delivered into onion epidermal cells using biolistic bombardment. YFP fluorescence was localized in the cytoplasm as in the case of Glyma.05g152100 (GmSHMT05c) and Glyma.08G187800 (GmSHMT08c), in the nucleus as in the case of Glyma.06g107800 (GmSHMT06n) and Glyma.08G187800 (GmSHMT08n), or presented a mitochondria-like subcellular localization in case of Glyma.02G217100 (GmSHMT02m) and Glyma.14G184500 (GmSHMT14m), or presented a chloroplastic-like subcellular localization in the case of the Glyma.13G222300 (GmSHMT13ch). No signal was detected in the case of the Glyma.15G089900 (GmSHMT15ch) (Corresponding to a truncated protein and is supposed to be a pseudogene). Bar = 100 µM.

To confirm *in silico* GmSHMT subcellular localization’s predictions, two cytosol-targeted *GmSHMT05c* and *GmSHMT08c*, two nucleus-targeted *GmSHMT06n* and *GmSHMT08n*, two mitochondrial-targeted *GmSHMT02m* and *GmSHMT14m*, as well as two chloroplastic-targeted *GmSHMT13ch* and *GmSHMT15ch* were studied for their subcellular localization by transforming onion epidermal cells using YFP fusions. The obtained results confirmed the subcellular localization predictions of the four GmSHMT classes. Indeed, GmSHMT05c::YFP and GmSHMT08c::YFP were localized in the cytosol of the transformed onion epidermal cells, whereas GmSHMT06n::YFP and GmSHMT08n::YFP accumulated in the nucleus (Fig. 3). *GmSHMT02m*::YFP presented a mitochondrial-like subcellular localization, while the GmSHMT14m::YFP presented both mitochondrial-like and cytosolic localization. The GmSHMT13ch::YFP presented a chloroplastic-like localization, and as expected, no signal has been detected for the GmSHMT15ch::YFP (coding for a truncated protein).

### Organ-specific expression of the *SHMT* genes

**Figure 4:**
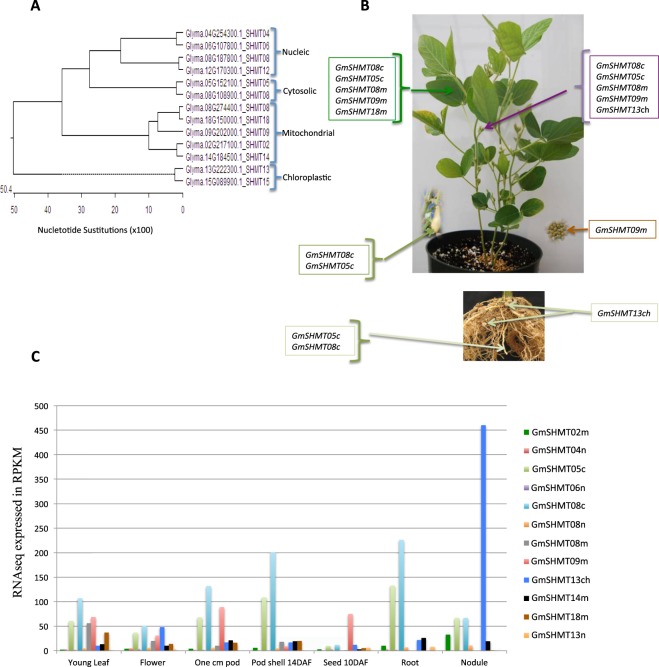
Expression patterns of the soybean *GmSHMT* gene members *in planta* were based on Soyseq resource available from RNAsequencing data (http://www.soybase.org/soyseq). **(A)** Phylogenetic tree of the 13 *GmSHMT* genes in soybean. **(B)** Tissue specific expression of the different *GmSHMT* classes. **(C)** RNAseq expression value of the different *GmSHMT* classes expressed in Reads/kilobase/million (RPKM) normalization of the raw data. No RNAseq expression data for *GmSHMT15ch* was available. No RNAseq expression data for *GmSHMT15ch* was available. GmSHMT15ch corresponds to a truncated protein and is supposed to be a pseudogene.

To gain insight into the expression profile divergence of different *GmSHMT* family members in soybean, expression data of each *GmSHMT* gene were compiled from the publically available RNA-seq database (Soybase.org)^34^. This dataset contains seven tissues from various developmental stages including vegetative (leaves, root and nodules) and seed development. While no RNAseq data were available for the *GmSHMT15ch*, the rest of the *GmSHMT* gene members presented different gene expression patterns (Fig. 4). Whereas most of the *GmSHMT* members presented an ubiquitous expression in all the tissues analyzed, the two duplicated cytosol-targeted *GmSHMT08c* and *GmSHMT05c* were highly expressed in roots and were predominantly expressed in pods. *GmSHMT08c*, *GmSHMT05c*, *GmSHMT08m*, *GmSHMT09m*, and *GmSHMT18m* were mainly expressed in young leaflets. *GmSHMT08c*, *GmSHMT05c*, *GmSHMT08m*, *GmSHMT09m*, and *GmSHMT13ch* were abundantly expressed in flowers.

*GmSHMT13ch* was highly abundant in the nodules. Expression data compiled from the public bio-analytic resource for plant biology database (http://bar.utoronto.ca) showed that the chloroplast-targeted *MtSHMT2ch* gene from *Medicago truncatula*, was abundantly expressed in leaves and seeds, but not in nodules, as was the case for the chloroplast-targeted *GmSHMT13ch* in soybean (Supplementary Fig. S6). Nucleus-targeted *MtSHMT2n* from *M*. *truncatula* was highly expressed in nodules^35^. Moreover, chloroplast-targeted SHMTs from the non-leguminous plant *Solanum lycopersicum* were abundantly expressed in fruit, leaves, and roots^36^. The chloroplastic *AtSHMT3ch* from *A*. *thaliana* was mainly expressed in seeds (Supplementary Fig. S6)^37,38^.

The mitochondria-targeted *GmSHMT09m* was mainly expressed in soybean seeds. Mitochondria-targeted *AtSHMT2m* from *A*. *thaliana* was also mainly expressed in seed, as well as in the root and 1^st^ node. However, the mitochondria-targeted MtSHMT5m from *M*. *truncatula* and the *SlSHMT2m* and *SlSHMT4m* from *S*. *lycopersicum* were mainly expressed in the pods/fruit, leaves, and roots (Supplementary Fig. S6). These data suggest that *GmSHMT* from the four different classes evolved to play different roles, and the function of a determined class is not organ specific.

### Expression analysis of the *GmSHMT* genes following soybean infection with SCN

**Figure 5:**
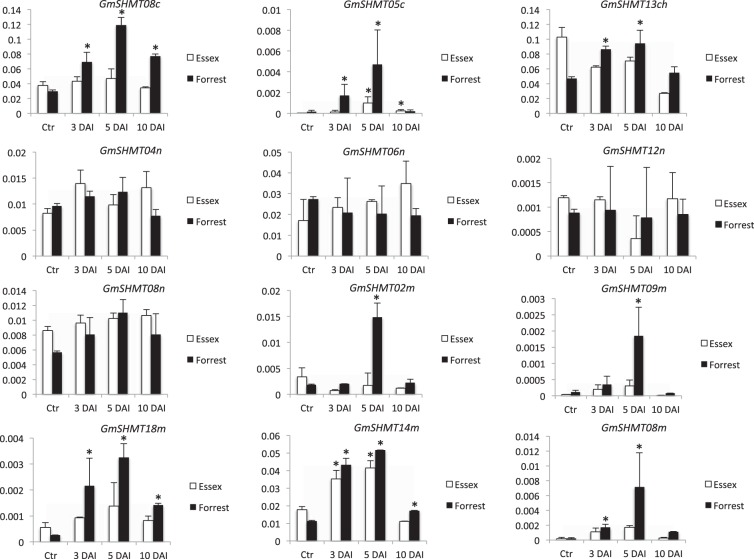
qRT-PCR of *GmSHMT* gene family in soybean in Forrest and Essex wild types. Quantitative RT-PCR analysis of the *GmSHMT* gene family members in chromosomes 04, 05, 06, 08, 09, 12, 14 and 18. The *GmSHMT15ch* gene in chromosome 15 was not expressed. Expressions were normalized using Ubiquitin as reference. (**E**) Essex, (**F**) Forrest, (**C**) without SCN infection, and (**D**) SCN infection at 3 and 5 days after inoculation. The gene-specific primers designed to amplify cDNA fragments are detailed in Supplementary Table S3. *Asterisks indicate significant differences between samples as determined by ANOVA (*P < 0.05). Error bars represent Standard deviations.

Unlike *GmSHMT* family members, the *GmSHMT08c* has been associated with SCN resistance^3^. To gain insight into the function of the *GmSHMT* family members, their specific response to SCN was investigated in the susceptible line Essex and the SCN resistant line Forrest (Peking-type; *Rhg4*a). Expression analysis using qRT-PCR at three, five, and ten days post SCN inoculation demonstrates that all cytosol, mitochondria, and chloroplast-targeted *GmSHMT* members were induced under SCN infection. Transcript abundance analyses showed that *GmSHMT* gene family members follow three types of expression patterns in response to SCN infection (Fig. 5). Whereas the cytosol-targeted *GmSHMT05c* and the mitochondria-targeted *GmSHMT14m* transcripts increased in both compatible and incompatible reactions to SCN, the chloroplast-targeted *GmSHMT13ch*, the cytosol-targeted *GmSHMT08c*, and the four mitochondria-targeted *GmSHMT02m*, *GmSHMT08m*, *GmSHMT09m*, and *GmSHMT18m* transcripts were significantly increased only in the incompatible reaction. However, transcripts from a third group including all the four nucleus-targeted *GmSHMT04n*, *GmSHMT06n*, *GmSHMT08n*, and *GmSHMT12n* were not induced during SCN infection (Fig. 5). The *GmSHMT15* gene was not expressed, which is coherent with the resuts obtained from the subcellular localization analysis (Fig. 3) and the RNAseq data (Fig. 4), suggesting that the *GmSHMT15* gene diverged from the others or has become pseudogenized.

### Domain variations within *GmSHMT* members

**Figure 6:**
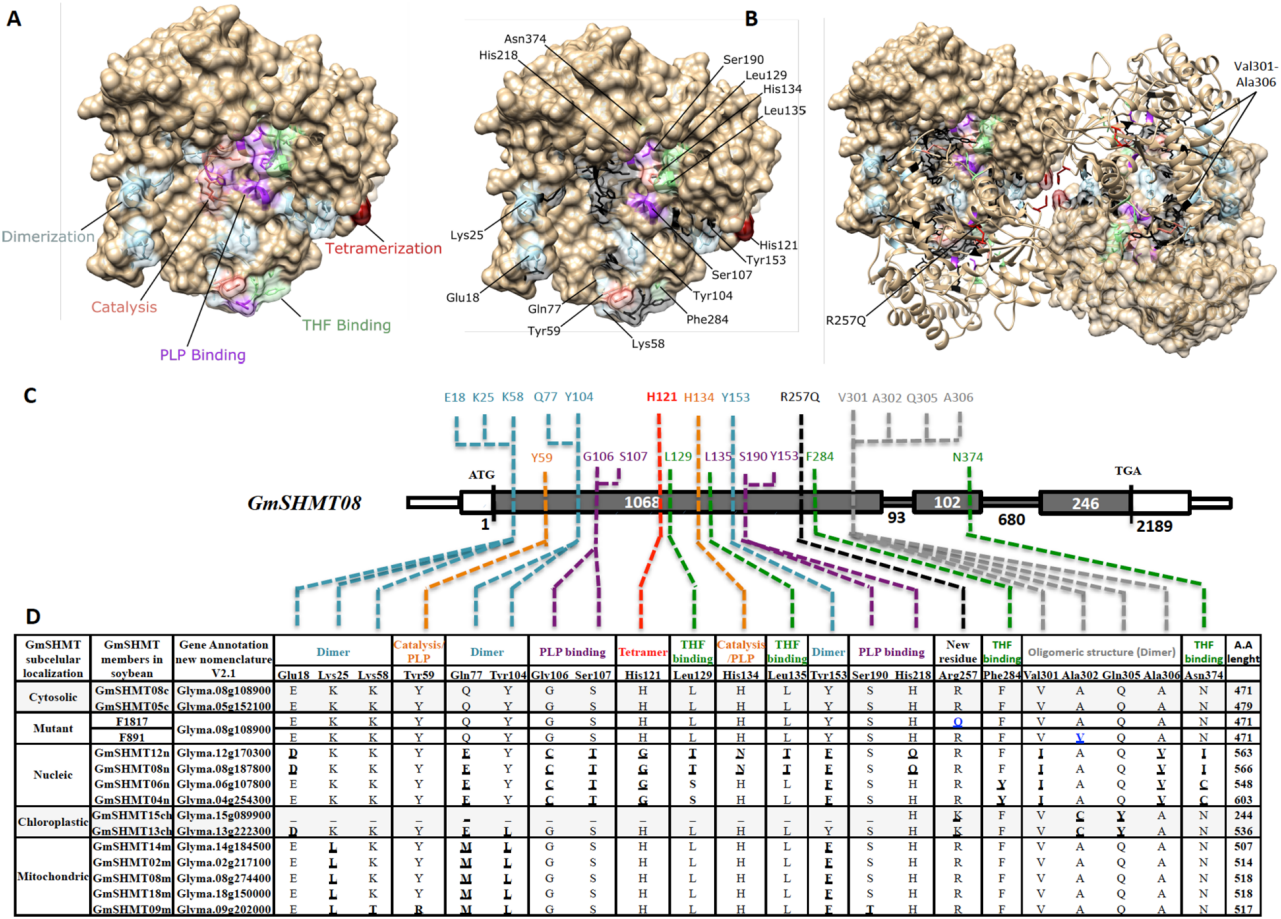
Homology modeling of the GmSHMT08c. (**A)** One SHMT subunit with highlighted catalytic sites, PLP and THF cofactor binding and oligomeric structural residues labelled. **(B)** Dimer with highlighted residues. **(C)** *GmSHMT08c* gene model showing the residues conserved for catalysis, PLP and THF cofactor binding, and oligomeric structure maintenance. **(D)** Polymorphisms presented by all the thirteen *GmSHMT* gene family members at important conserved residues cited previously.

Investigation of GmSHMT08c for conserved domain showed the presence of forty amino acid residues that play an important role in maintaining dimer interfaces (16 residues), tetrahydrofolate (THF) binding sites (7 residues), pyridoxal phosphate (PLP) cofactor binding sites (13 residues), and active sites for catalysis (4 residues) (Fig. 6A,B)^39^. *In silico* analysis showed that the cytosolic GmSHMT05*c* member presents the same conserved residues like GmSHMT08c (Fig. 6C), confirming their segmental duplication inferred from syntenic analysis. However, nucleic, cytosolic, and mitochondrial-targeted GmSHMTs show variations within their oligomeric structures, catalytic sites, and cofactor binding residues (Fig. 6D).

Nucleic GmSHMT members did not conserve most of the residues at positions Q77, G106, S107, H121, L129, Y153, V301, A306, and N374. In addition, E18, H134, and L135 residues differ in GmSHMT08n and GmSHMT12n, whereas the F284 residue was not conserved in both GmSHMT04n and GmSHMT06n. Moreover, Q77, Y104, R257, A302, and Q305 residues were not conserved in all chloroplastic-targeted GmSHMTs.

Mitochondrial-targeted GmSHMTs did not conserve K25, Q77, Y104, and Y153 residues. The GmSHMT09m gene family member had differing residues from the two cytosolic-targeted GmSHMTs at positions K58, Y59, S190, and G310 (Fig. 6D).

Domain variation analysis of the GmSHMT classes showed that the highest domain variations were observed within the nucleic-targeted GmSHMT classes, with 14 domain variation out of 40 (35%), affecting protein structure (dimerization and tetramerization), substrate binding (including THF and PLP binding), and catalysis (Figs 1 and 6). However, less domain variation was observed in the mitochondrial-targeted GmSHMT classes; 6 out of 40 (15%), suggesting functional conservation during evolution. Similarly, the mitochondrial-targeted GmSHMT class presented limited variations; 4 out of 40 (10%), except for the GmSHMT09m that conferred extra 3 domains variation (17.5%). As expected, no domain variation has been observed between the two cytosolic-targeted GmSHMT05c and GmSHMT08c.

### SCN susceptible EMS-induced mutant lines carry mutations at the *GmSHMT08c* only

**Figure 7:**
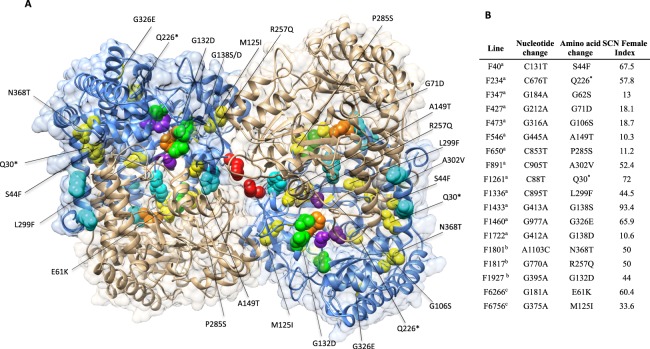
The identified eighteen EMS *Gmshmt08c* mutants. Homology modeling of a GmSHMT08c asymmetric homotetramer predicted protein structure, showing all the eighteen identified *Gmshmt08c* mutants, in addition to important GmSHMT08c residues. Four subunits are shown, representing a GmSHMT08c homomer each; (**A**) GmSHMT08 homomer upper right, (**B**) GmSHMT08 homomer down right, (**C**) GmSHMT08 homomer upper left, (**D**) GmSHMT08 homomer down left. Only GmSHMT08c subunits (**B**,**C**) were highlighted with PLP/catalytic sites (orange), PLP cofactor binding (purple), THF cofactor binding (green), oligomeric structure maintenance (gray), dimerization (cyan), and tetramerization (red) residues. All eighteen *Gmshmt08c* mutants were mapped, represented in yellow sphere, and labeled in the subunits (**B**,**C**). All the susceptible EMS mutagenized Forrest mutants that were identified by forward genetics presented mutations at the *GmSHMT08c* gene only. ^a^Mutants identified by forward genetics (Kandoth *et al*.^40^). ^b^Mutants identified by forward genetics in the current study, ^c^Mutants identified by TILLING (Liu *et al*.^3^).

To gain more insight into the possible involvement of the *GmSHMT* gene family members in resistance to SCN, we developed EMS mutagenized soybean lines from the SCN resistant Forrest. Next, forward genetic screening was employed to screen for mutants that lost their resistance to SCN. The susceptible Forrest EMS mutagenized population was screened for mutations within all the *GmSHMT* gene family members. In the current study, three new EMS mutants were found to lose their resistance to SCN, in addition to the fifteen EMS mutants that we reported recently^3,40^. Targeted sequencing analysis showed that all eighteen mutations were located in the *GmSHMT08c* gene. Interestingly, none of the selected *Gmshmt08c* mutants carried mutations in the coding or non coding regions of the other 12 *GmSHMT* genes corresponding to the four classes including the cytosolic-targeted *GmSHMT05c* member. In total, sixteen *Gmshmt08* missense mutants were identified. The *Gmshmt08* EMS mutants F6266 (E61K) and F6756 (M125I) were identified earlier by reverse genetics^3^, F427 (G71D), F1336 (L299F), F891 (A302V), F1460 (G326E), F1433 (G138S), F1722 (G138D), F473 (G106S), F40 (S44F), F347 (G62S), F546 (A149T), F650 (P285S) were reported recently^40^, in addition to the newly isolated *Gmshmt08* EMS mutants F1801 (N368T), F1927 (G132D), and F1817 (R257Q) were identified by forward genetics. Two additional *Gmshmt08* nonsense mutants F1261 (Q30*) and F234 (Q226*) have been identified to carry a premature stop codon after 30 and 226 amino acid of the GmSHMT08 protein, respectively. The presence of the new induced GmSHMT08 haplotypes resulted in the increase of SCN female indexes in all the identified missense mutants, up to 93.4%, indicating that Forrest mutants became susceptible to SCN, although these *Gmshmt08* EMS mutants do not carry any mutations on the other 12 *GmSHMT* genes (Fig. 7). Thus, none of the other *GmSHMT* gene family could replace the function of the *GmSHMT08c* in all the identified EMS mutagenized *Gmshmt08c* mutants. These findings support the hypothesis of the absence of functional redundancy among the *GmSHMT* gene family in resistance to SCN.

### SNP variants and haplotype analysis of the *GmSHMT* gene family members

The Soybase database provides a beneficial integrated genetic linkage map tool that can be used to infer whether a gene may belong to a given QTL (Cregan *et al*., 1999). In the last two decades, 189 SCN QTLs have been mapped and reported in the soybean genome, distributed across 19 out of 20 soybean chromosomes. Extensive search of the *GmSHMT* genes showed that *GmSHMT08c* had been consistently mapped across a variety of soybean germplasm and represented the major source of resistance in most soybean cultivars at the *Rhg4* locus^2,3,41^. Beside the *GmSHMT08c*, the other cytosol-targeted paralog *GmSHMT05c*, in addition to the other three mitochondria-targeted *GmSHMT09m*, *GmSHMT08m*, and *GmSHMT18m* have also been found in a QTL for resistance to SCN^42^. However, all four nucleic and chloroplast-targeted *GmSHMT*13, in addition to the other two mitochondria-targeted *GmSHMT14m* and *GmSHMT02m* were mapped in QTL that were linked to other agronomic traits including seed content and composition (oil, protein, etc.), seed yield, and plant development, but none of those have been found within QTL for resistance to SCN (Table 1).

**Table 1:** Summary of the *GmSHMT* gene family members and their corresponding identified QTLs mapped in soybean (Soybase database).

Gene name	Gene ID	Gene position	QTL	QTL position	Parents	Number loci tested	Lod score	Interval length	Reference
GmSHMT08c	Glyma.08g108900	Gm08: 08,358,422 – 08,363,343	SCN	Gm08: 03,828,727 − 08,388,481	Magellan X PI 404198A	194	5.8	25.6	Guo *et al*., 2006
GmSHMT05c	Glyma.05G152100	Gm05: 34,563,195 – 34,565,889	SCN	Gm05: 14,157,044 – 35,074,014	PI438489B X Hamilton	115	ND	18.3	Yue *et al*., 2001
GmSHMT13ch	Glyma.13G222300	Gm13: 33,522,900 – 33,527,302	Seed protein	Gm13: 31,220,086 – 38,929,324	Essex X Williams	ND	ND	ND	Hyten *et al*., 2004
GmSHMT04n	Glyma.04G254300	Gm04: 48,571,800 – 48,577,505	Seed weight	Gm04: 48,708,390 – 52,389,145	Charleston X Dong Nong 594	ND	ND	2	Teng *et al*., 2009
GmSHMT06n	Glyma.06G107800	Gm06: 08,663,690 – 08,668,262	Pod maturity	Gm06: 07,057,089 – 08,964,865	Minsoy X Noir 1	665	ND	ND	Specht *et al*., 2001
GmSHMT12n	Glyma.12G170300	Gm12: 32,509,864 – 32,514,684	Neutral detergent fiber	Gm12: 32,410,129 – 35,695,155	PI 483463 X Hutcheson	ND	10.48	ND	Asekova *et al*., 201
GmSHMT08n	Glyma.08g187800	Gm08: 15,060,906 – 15,065,634	Seed genistein	Gm08: 04,776,921 – 20,678,814	Zhongdou 27 X Jiunong 20	606	ND	73.1	Han *et al*.,. 2015
GmSHMT02m	Glyma.02g217100	Gm02: 40,402,376 – 40,409,818	First flower	Gm 02: 31,189,638 – 41,513,786	JP036034 X Ryuhou	720	ND	13.85	Kuroda *et al*., 2013
GmSHMT09m	Glyma.09g202000	Gm09: 42,616,719 – 42,623,320	SCN	Gm09: 41,477,149 – 47,446,419	S08-80 X PI 464925B	118	2.08	24.67	Winter *et al*., 2007
		Gm09: 42,616,719 – 42,623,320	Pod number	Gm09: 36,924,281 – 45,989,139	BARC-8 X Garimpo	75	2.06	24.5	Vieira *et al*., 2006
GmSHMT08m	Glyma.08g274400	Gm08: 36,279,886 – 36,286,158	SCN	Gm08: 16,438,021 – 40,476,678	Magellan X PI 567516 C	252	4.3	18.55	Vuong *et al*., 2010
		Gm08: 36,279,886 – 36,286,158	Seed oil plus protein	Gm08: 20,683,774 – 47,837,939	Charleston X Dong Nong 594	164	2.6	38	Chen *et al*., 2007
GmSHMT18m	Glyma.08g274400	Gm18: 27,237,675 – 27,243,626	Seed glycinin	Gm18: 20,151,954 – 48,589,101	Kefeng No. 1 X Nannong 1138-2	221	ND	11.11	Ma *et al*., 2016
		Gm18: 27,237,675 – 27,243,626	SCN	Gm18: 03,729,034 – 50,158,095	PI438489B X Hamilton	115	ND	26.1	Yue *et al*., 2001
GmSHMT14m	Glyma.08g274400	Gm14: 44,788,273 – 44,795,824	Fe effic	Gm14: 40,368,007 – 47,207,943	Pride B216 X A15	102	2.7	14	Lin *et al*., 1997

**Figure 8:**
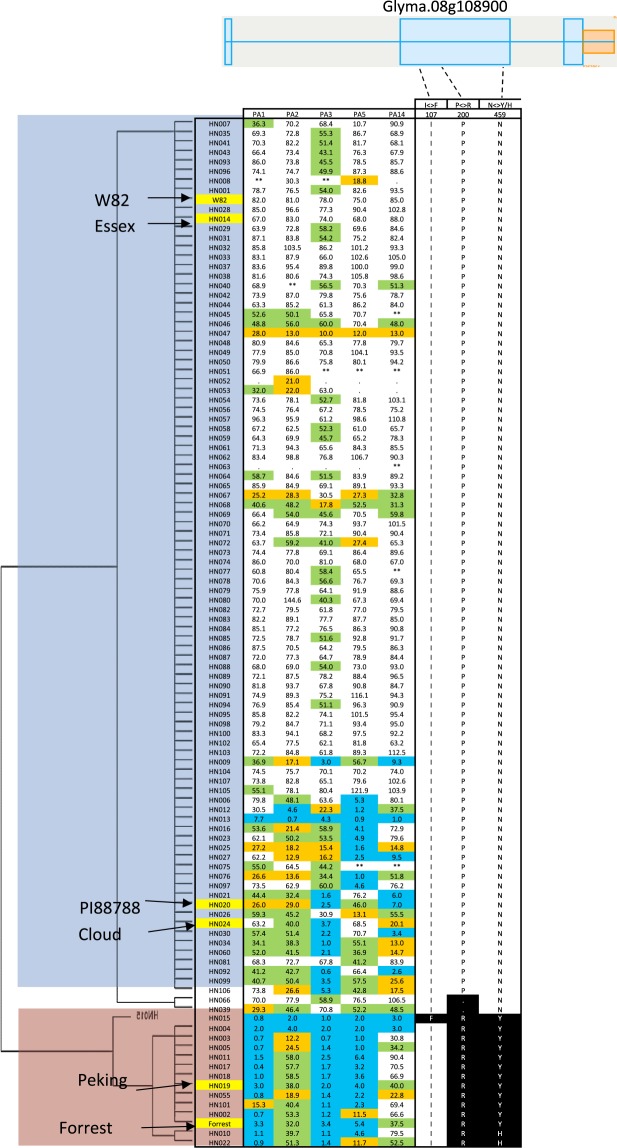
Haplotype clustering and correlation with soybean cyst nematode female index of the cytosolic-localized *GmSHMT08c* in the 106 soybean lines. The 106 soybean lines included non-domesticated; semi-domesticated, and elite domesticated introductions belonging to the USDA soybean collection. Schematic graph shows the position of SNP/indel for *Glyma*.*08g108900* (*GmSHMT08c*) gene. SNP in black background were specific to Peking-type of resistance, and clustered with soybean lines carrying resistance to three SCN Hg-types; 0, 2.7, and 2.5.7. Blue box represents exon, blue bar represents intron, orange box represents promoter region, and grey box represents 3′ or 5′ UTR. SNPs were positioned relative to the genomic position in W82. SNP position in red text showing non-synonymous SNPs leading to amino acid change. Female Index in blue (FI < 10, resistant to SCN), in green (10 < FI < 30, Moderate resistance), in orange (30 < FI < 60, Moderate susceptibility), in white (60 < FI, Susceptible to SCN).

Haplotype diversity at the *Rhg4* loci determines the SCN resistance at different re-sequenced soybean germplasms^3^. In fact, only lines carrying H1, H2, and H3 haplotypes at the *GmSHMT08c* have been shown to be resistant to SCN^3^. However, soybean lines carrying other GmSHMT08 haplotypes (i.e. H4, H5, and H8) were susceptible to SCN. The sequence diversity and identification of these *Rhg4* haplotypes from a wide range of soybean belonging to the USDA soybean germplasm collection would be beneficial for breeders. In the current study, the possible role of the *GmSHMT* members in SCN resistance was explored using the natural variations in *GmSHMT* genes. Correlation with SCN resistance to the five SCN races using whole genome resequencing data (WGRS) was used. To infer the allelic variation in 106 diverse soybean lines^43^, all *GmSHMT* genes were analyzed for synonymous and non-synonymous SNPs; premature stop codons; and indels. The WGRS dataset included non-domesticated; semi-domesticated, and elite domesticated introductions belonging to the USDA soybean collection. Interestingly, haplotyping analysis of the 106 lines showed that none of the *GmSHMT* gene family members presented specific *GmSHMT* haplotypes that correlate with SCN resistance (Supplementary Figs S7–S17), with the exception of the *GmSHMT08c* (Fig. 8). In fact, only *GmSHMT08c* presented two non-synonymous SNPs, resulting in proline to arginine (P130R) and asparagine to tyrosine/serine (N358Y/H) changes in 14 out of 106 lines analyzed (Fig. 8). All 14 soybean lines (belonging to Peking-type of SCN resistance) carrying these non-synonymous SNPs at *GmSHMT08c* correlated with resistance to SCN.

## Discussion

### *GmSHMT* family evolved in early land plant evolution through genome duplication and divergence

The soybean genome contains a high number of *SHMT* genes (about 18 members), compared to the plant model *A*. *thaliana* (7 members), *S*. *lycopersicum* (7), or *M*. *truncatula* (12) (Supplementary Table S2). Previous studies suggested that segmental duplications, tandem rearrangement, and polyploidy events contributed to their evolution^20,21,23,44^. The conservation of duplicates is likely the result of functional divergence through sub-functionalization and neo-functionalization under selection pressure imposed by developmental and environmental conditions.

Phylogenetic analyses showed that land plants have four subclasses of *GmSHMT*s, reflecting their subcellular distribution (cytosol, nucleus, mitochondrion, and chloroplast), which is in agreement with a previous study^18^. Subcellular localization of selected GmSHMT members confirmed their *in-silico* prediction and phylogenetic distribution. The existence of *SHMT* sequences representing all classes in *C*. *reinhardtii* suggests that at least three *SHMT* classes (mitochondria, chloroplast, and a common nucleus/cytosol) evolved early in the ancestor of land plants through duplication and functional divergence. The presence of a single *C*. *reinhardtii* (*Cre06*.*g293950*) *SHMTn/c* sequence clustering with nucleus and cytosol *SHMT* suggests that *SHMT* from these classes evolved from a single ancestor gene through duplication. An alternative explanation is that one of the copies may get lost after duplication. In addition, the phylogenetic distribution of sequences from the same species suggests that duplication/polyploidization events occurred at the species level and contributed to the duplication of *SHMT* genes. This is in accordance with previous studies reporting large segmental duplications or whole genome duplication in various plant species^45–47^.

Structural analysis revealed various patterns of intron/exon loss or gain that may happen during the evolution of the *SHMT*. It has been reported earlier the presence of an intron loss that occurred within the cytosolic *SHMTc*^18^. The current study reveals the presence of several intron loss/gain events that may have occurred early in land plant ancestor.

It is noteworthy that no chloroplast-targeted SHMTs exist in monocotyledon genomes. This is consistent with previous studies reporting the absence of the SHMT activity in wheat chloroplasts^14^. Chloroplastic SHMT is essential in providing one-carbon units for the biosynthesis of 5,10-methyleneTHF, which is then oxidized by other catalysts^9,48,49^. The oxidized 5,10-methyleneTHF is essential as a light-harvesting cofactor in plastid-localized cryptochrome^50^. It’s well documented that the products of photosynthesis feedback into the circadian clocks to set the plant’s rhythm, increase the photosynthesis rate, growth, survival, and competitiveness advantage^51–53^. This process helps plants regulate physiology and triggers metabolism and stress responses. Recent studies have shown that the circadian clock’s effect on the daily growth rhythms is different between monocots and eudicots^54,55^. We speculate that the absence of chloroplastic GmSHMTs in monocots could be linked to these reported differences between monocots and eudicots. However, this hypothesis needs further investigation.

### Domain variation suggest the presence of altered affinities in nucleic, chloroplastic, and mitochondria targeted GmSHMTs

Structural analysis of the *GmSHMT* gene family members showed that the four nucleic GmSHMTs did not conserve the same residues involved in subunit assembly, THF and PLP binding if compared to the cytosolic GmSHMTs (Figs 1B and 6D). For instance, it was inferred that the nucleus-targeted GmSHMT may have acquired new substrate affinities, lost their PLP cofactor binding, catalysis, and/or subunit assembly (tetramerization), as it was shown using induced mutagenesis within the H134N, H147N, and H150N mutants of sheep liver serine hydroxymethyltransferase (ScSHMT) corresponding to His121 and His134 residues in soybean GmSHMT08^56^. Interestingly, both spontaneously occurring mutations at His121 and His134 were found to be located four, two, and four amino acids away from the identified M125I, G132D, and G138S/G138D mutations at the *GmSHMT08c* gene, respectively. These two mutants from a mutagenized EMS Forrest soybean population lost their resistance to SCN.

Moreover, the Gln77 and Tyr104 residues involved in SHMT dimer interface were not conserved in the chloroplast-targeted GmSHMTs; in addition to the Ala302 and Gln305, which have been demonstrated to be essential for maintaining the SHMT oligomeric structure^57,58^. Gln77 and Tyr104 natural occurring mutations are located six and two residues away from G71D and G106S *Gmshmt08c* EMS induced mutants, respectively. Ala302 and Gln305 residues were found to be located three and six residues away from another GmSHMT08c L299F EMS induced *Gmshmt08c* mutant. Interestingly, all three reported mutations in the resistant Forrest soybean lost their SCN resistance. The *Gmshmt08c* A302V mutant which corresponds to the Ala302 increases also susceptibility to SCN up to 52.4%. Chloroplastic GmSHMT13 and GmSHMT15 did not conserve Arg257 residue. The Arg257 is important for maintaining SCN resistance in soybean since we observed that the induced EMS R257Q mutation at the *GmSHMT08c* increases susceptibility to SCN up to 50%.

Furthermore, Lys25, Gln77, Tyr104, and Tyr153 residues involved in SHMT dimer interface were different among the mitochondria and cytosol-targeted GmSHMTs. Tyr153 is found two residues away from the A149T *Gmshmt08c* mutation, which may have increased SCN resistance up to 10.3%. The mitochondrial GmSHMT09 member did not present the same Tyr59 and Ser190 conserved residues, both involved in PLP binding and catalysis. Tyr59 residue is located two amino acids away from the EMS induced E61K *Gmshmt08c* mutant, which increases SCN susceptibility up to 60%.

Additionally, *in vitro* kinetic studies of *Gmshmt08c* mutated alleles when compared to the Forrest *GmSHMT08c* allele, used as a positive control, showed differences in enzymatic activity of the GmSHMT08c protein^3,40^. *GmSHMT08c* alleles carrying the E61K, G71D, G326E, A302V, and M125I mutations resulted in proteins that are enzymatically inactive, as they were unable to support the growth of bacteria. In contrast, the *GmSHMT08*c allele from the wild type Forrest supported growth of the mutant bacteria^3,40^.

Therefore, we hypothesize that the accumulation of these natural spontaneous mutations may alter the function and/or oligomeric structure of the nucleus, mitochondria, and chloroplast-targeted GmSHMT proteins, or may alter their substrate affinities, ultimately leading to their sub-functionalization or/and neo-functionalization contributing to the loss of SCN resistance and gain of new functions^3,40,59^. Thus, modeling of the GmSHMT enzyme structures of all soybean isozyme classes presented features that may explain the differences in activity and SCN resistance between the GmSHMT classes.

### Soybean *GmSHMT* genes display overlapping responses and divergent functions in resistance to SCN

All four nucleus-targeted *GmSHMT* members were not induced under SCN infection. Protein modeling showed that most of the domain variations occurred within the nucleus-targeted *GmSHMT* members only. Thus, we suggest that the high number of domain variations may alter the enzymatic activity and change the affinity of the protein and thus its function toward SCN resistance. Although cytosol, chloroplast, and mitochondria-targeted *GmSHMT* members were induced in SCN incompatible interaction; only the cytosolic *GmSHMT08c* has been shown to be involved in resistance to SCN. This was observed within the soluble NSF attachment protein (*GmSNAP*) gene family, where only the *GmSNAP18* was the major gene for SCN resistance. In fact, although four *GmSNAP* gene members were induced in response to SCN inoculation, *GmSNAP14* and *GmSNAP02* were not involved in SCN resistance, but only *GmSNAP18* and *GmSNAP11*^19^. Although the last duplications happened about 13 million years ago^23^, *GmSHMT* genes have likely accumulated mutations that led to the divergence of their protein sequences, ultimately leading to a neofunctionalization or subfunctionalization.

Despite the observed divergence in the structure and function within the cytosol, chloroplast, and mitochondria-targeted *GmSHMT* gene family, the overlapped expression of certain members is most likely due to their response to the same regulatory elements. In fact, although different members of the *GmSHMT* family have evolved to mediate different functions due to the presence of natural occurring mutations, certain members are still responding to the same stimuli. Similar functional patterns have been observed within the orthologs of the soybean *SWEET* gene family, which acquired new functions during evolution^60^. Whereas some *SWEET* genes have been involved in seed development, other *SWEET* members play a major role in disease resistance. Furthermore, it has been reported that duplications resulting in multiple gene families represent functional redundancy and/or divergence^19,61,62^. Previous studies in *Arabidopsis* have shown that three members (TTL1, TTL3, and TTL4) of the Tetratricopeptide repeat thioredoxin-like (TTL) gene family are required for osmotic stress tolerance presenting an additive effect and are essential for root growth and integrity^61^. However, the TTL2 member diverged and acquired a novel function in male sporogenesis. This is a good case of gene functional redundancy and gene divergence that occurred within the same gene family. It has been reported that duplication of the *CLAVATA* gene family follows a process of divergence in the function of their gene members. In fact, *GmCLV1A* acts on shoot architecture, whereas *GmCLV1B* (*GmNARK*) functions in controlling nodule numbers^63^. In the current study, characterization of the *GmSHMT* members demonstrates that this gene family diverged over time and gave rise to different *GmSHMT* members with the absence of functional redundancy in resistance to SCN.

This characteristic has been supported by the SNP variant analysis of the *GmSHMT08c* that was extended to the rest of the *GmSHMT* gene family members. In fact, only *GmSHMT08c* member presented distinct haplotype formed by these SNPs which correlates with resistance and susceptibility to SCN within the 106 soybean lines analyzed. All 14 lines belonging to Peking-type of resistance, which requires in addition to the *rhg1*-a (*GmSNAP18*) the presence of the *Rhg4* locus (*GmSHMT08c*), presented different *GmSHMT08*c haplotype from the susceptible lines that cluster with SCN resistance (Fig. 8). The rest of the lines showing resistance to SCN but carrying susceptible haplotype at the *Rhg4* locus (*GmSHMT08*c) could be explained by their PI88788 source of resistance that does not require the resistant *Rhg4* allele (*GmSHMT08*c); and thus, their resistance is due to the presence of the *rhg1-b* locus only (Fig. 8), which haplotype has been shown to be different than the rest of soybean SCN susceptible lines^3,4^.

Although the role of the other *GmSHMT* members needs to be elucidated, this study revealed that *GmSHMT* genes might have diverged or acquired new function during the evolution. While several members showed an overlap in their responses to SCN stimulus, only *GmSHMT08c* function in resistance to SCN. The functional divergence of *GmSHMT* genes had likely happened through exon skipping, alternative splicing variants, amino acid polymorphism, organ-specific expression, and subcellular localization. Considering the function of soybean *GmSHMT08* in SCN resistance, the duplication and retention of *SHMT* genes in plants suggest that *SHMT* genes may play a key role in soybean adaptation, which is important for proper responses to changing environments.

## Material and Methods

### SHMT sequences and phylogenetic analysis

SHMT sequences used in phylogenetic analyses include sequences retrieved from different databases including NCBI, Soybase (W82.a2.v1), and Phytozome (v12.1). Sequences were identified by querying Arabidopsis sequences against sequences from these databases employing tblastn using default parameters. The retrieved sequences were checked for motifs (PLP, THF, catalysis, dimerization, and tetramerization) that are specific to this family. Only sequences with similarity to *GmSHMT* genes that have the previously mentioned motifs were considered in this study. *GmSHMT* gene organization (introns and exons), and their predicted amino acid sequences were retrieved from the Phytozome database (v12.1). *GmSHMT* genomic and corresponding protein sequences from the first splicing presenting the translated amino acid sequences from all the exons have been used in the analyses. We used sequences from sets of plants with fully sequenced genomes representing key positions on the angiosperm phylogenetic tree. Sequences were carefully inspected and corrected for annotation errors before use. The analysis included the GmSHMTs identified in soybean, in addition to SHMTs from eudicot species including the seven cytosolic, mitochondrial, chloroplastic, and nucleic SHMTs from the eudicot model (*A*. *thaliana*), other monocots including a monocot model (*O*. *sativa*), and the most primitive lineage models including a lycophyte (*S*. *moellendorfii*), a moss (*P*. *patens*), and a chlorophytic algae (*C*. *reinhardtii*).

SHMT nucleotide cDNA sequences were translated into protein sequences. The inferred protein sequences were then aligned using Muscle with default parameters, and manually adjusted. Phylogenetic analyses were performed on the aligned amino acid sequences, in PHYML using the WAG model and assuming among site rate heterogeneity (WAG + G). 1000 bootstrap replicates were run to estimate branch support.

### *GmSHMT* cloning and subcellular localization

The coding sequences of the *GmSHMT* genes (Glyma.02G217100, Glyma.05g152100, Glyma.08G108900, Glyma.06g107800, Glyma.08G187800, Glyma.13G222300, Glyma.14G184500, and Glyma.15G089900) were amplified from Forrest cDNA using forward and reverse primers containing *EcoRI* or *Hind*III and *Sal*I restriction enzyme sites, respectively. PCR products were digested and then ligated to the N-terminus of the yellow fluorescent protein (*YFP*) reporter gene in the *pSAT6-EYFP-N1* vector. The fusion constructs were verified by sequencing. Gold particles were coated with plasmid DNA and delivered into onion epidermal cells using biolistic bombarded as previously described^47^. The bombarded onion epidermal peels were kept in the dark at 26 °C for at least 20 hours before being visualized using EVOS® FL Auto Cell Imaging System (Life Technologies) to determine the subcellular localization of the fused proteins.

### qRT-PCR analysis of *GmSHMT* gene family members

Soybean seedlings from the SCN susceptible line Essex and from the SCN resistant line Forrest were grown in autoclaved silt loam and sandy soil in the growth chamber for one week and then infected with eggs from the PA3 population. Total RNA was isolated from root samples after three, five, and ten days following SCN infection as described previously^19^. Experiments were repeated threefold with similar results. Results from one biological replicate are shown. All presented results were performed with the analysis of variance ANOVA, using JMP Pro V12 software as described earlier^19,64^. Primers used for qRT-PCR are listed in Supplemental Table S3.

### Homology modeling of GmSHMT08c and mutational analysis

Homology modeling of a putative asymmetric homotetrameric SHMT protein structure was conducted with Deepview and Swiss Model Workspace software using the SHMT protein sequence from Forrest and the available SHMT crystal structure from *Homo sapiens* as a template; PDB accession 1BJ4 chain A^65^. All THF and PLP binding sites, active sites, and catalysis residues retrieved from NCBI conserved domain database were modeled against this template with a sequence identity of 60%^59^. *GmSHMT* EMS induced and natural mutations and haplotypes identified were then mapped onto the model.

### Development of the EMS mutagenesis Forrest population

The soybean *c*.*v*. Forrest seed was from Southern Illinois University-Carbondale Agricultural Research Center, and was used to develop an EMS mutagenized population. The wild type Forrest seed was mutagenized with 0.6% (w/v) EMS as described by^66^, and planted to harvest 1,536 and 2,827 M2 families of seed in 2011 and 2013 respectively, which was then advanced to M3 generation in 2014.

### SCN-infection phenotyping and forward genetics screeening

SCN-infection screening was performed on the EMS induced Forrest mutants, as described by^67^. More than 3,000 mutant families from both 2011 and 2013 M2 generations were screened for SCN resistance. Eighteen EMS mutants identified from Forrest lost their resistance to SCN. All eighteen mutants carried missense (16 mutants) or nonsense (2 mutants) mutations at the *GmSHMT08c*, but not on the rest of the *GmSHMT* members.

### Haplotype clustering analysis

The whole genome re-sequencing data^43^ of 106 diverse soybean lines (15X coverage) was utilized to identify allelic variants and haplotype in *GmSHMT* gene family. SNP based haplotypes were examined by generating map and genotype data files using TASSEL 5.0 program and clustering pictorial output for SACPD gene. The clusters were created and visualized using FLAPJACK software^68^ as described in details previously^69^. Additionally, the phenotypic data for soybean cyst nematode screening was obtained from Nguyen Lab (unpublished) and clustered with SNP matrix. Transcript sequence-based annotation (W82.a2. v1) was used to classify synonymous and non-synonymous SNPs by translation into amino acid sequences.

## Supplementary information

Supplementary Figures

## Data Availability

The developed EMS mutagenized Forrest mutants are the property of Southern Illinois University but released to all requestors. The new serine hydroxylmethyltransferase (SHMT; EC 2.1.2.1) alleles are deposited at NCBI. GenBank accession numbers are listed in Supplemental Table S4.
