# Supplementary material for: Genome reorganization of the GmSHMT gene family in soybean showed a lack of functional redundancy in resistance to soybean cyst nematode
Source: Sci Rep. 2019 Feb 6;9:1506. doi: 10.1038/s41598-018-37815-w (PMC6365578; doi:10.1038/s41598-018-37815-w)
Supplement: Supplementary file 1 — Supplementary Figures [file 41598_2018_37815_MOESM1_ESM.pdf]

Genome reorganization of the *GmSHMT* gene family in soybean showed a lack of functional redundancy in resistance to soybean cyst nematode

Naoufal Lakhssassi[1], Gunvant Patil[2,3]*, Sarbottam Piya[4*], Zhou Zhou[1*], Azam Baharlouei[1], My Abdelmajid Kassem[5], David A Lightfoot[1], Tarek Hewezi[4], Abdelali Barakat[6], Henry T. Nguyen[2], and Khalid Meksem[1]

[1]Department of Plant, Soil and Agricultural Systems, Southern Illinois University, Carbondale, IL 62901, USA. [2]Division of Plant Sciences, University of Missouri, Columbia, MO, 65201, USA. [3]Department of Agronomy and Plant Genetics, University of Minnesota, St. Paul, MN 55108, USA. [4]Department of Plant Sciences, University of Tennessee, Knoxville, TN, 37996, USA. [5]Department of Biological Sciences, Fayetteville State University, NC 28301, USA. [6]Department of Biology, University of South Dakota, Vermillion, SD 57069, USA.
*These authors contribute equally to this work. Correspondence to meksem@siu.edu.

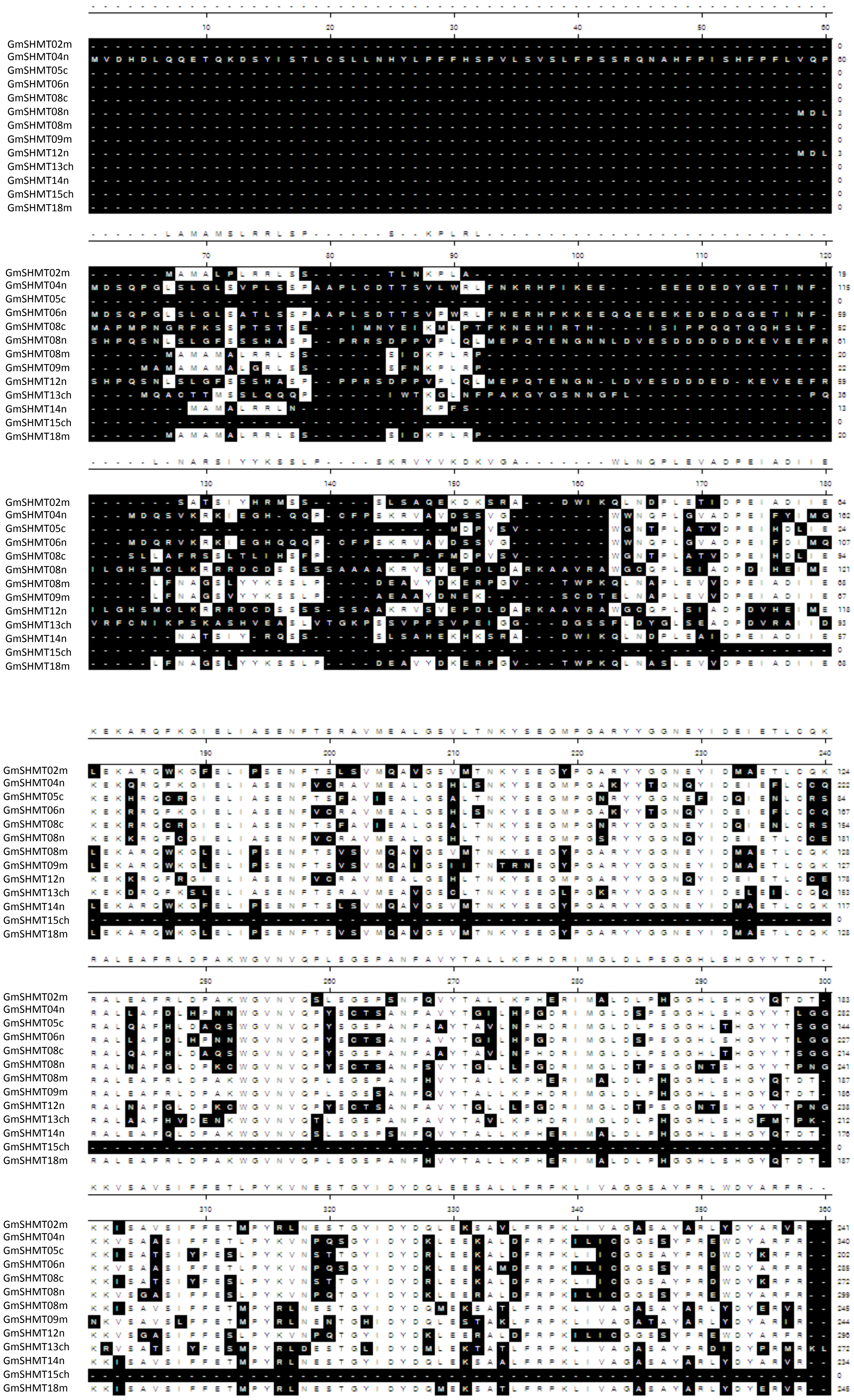
GmSHMT02m
GmSHMT04n
GmSHMT05c
GmSHMT06n
GmSHMT08c
GmSHMT08n
GmSHMT08m
GmSHMT09m
GmSHMT12n
GmSHMT13ch
GmSHMT14n
GmSHMT15ch
GmSHMT18m

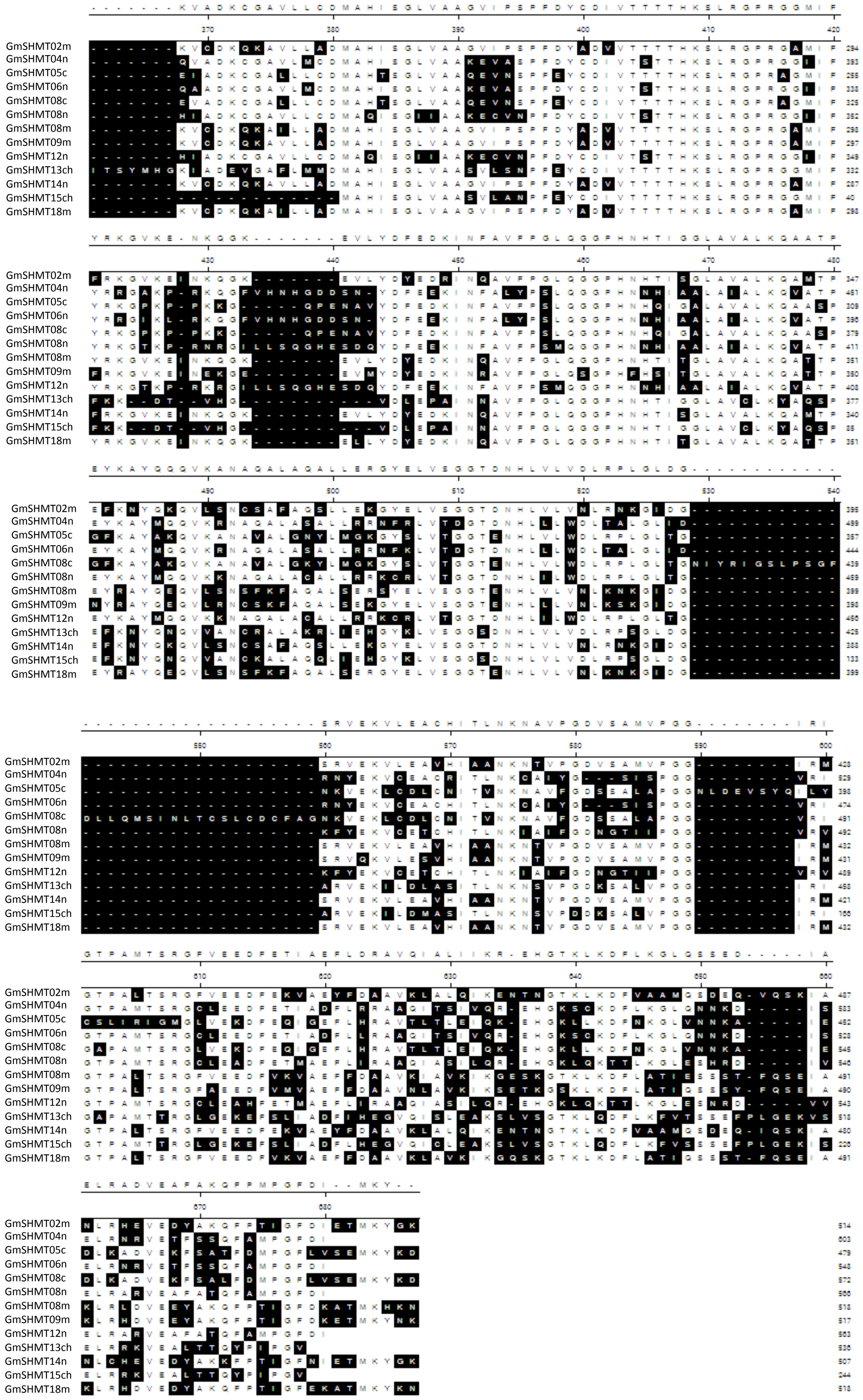

Supplementary Figure S1. Comparative analysis of GmSHMT proteins in soybean. Amino acid alignment for the predicted GmSHMT protein members in soybean. *In silico* analysis was performed using the MegAlign (DNASTAR Lasergene 8) software package and the Clustal W algorithm. All parameter values correspond to default definitions.

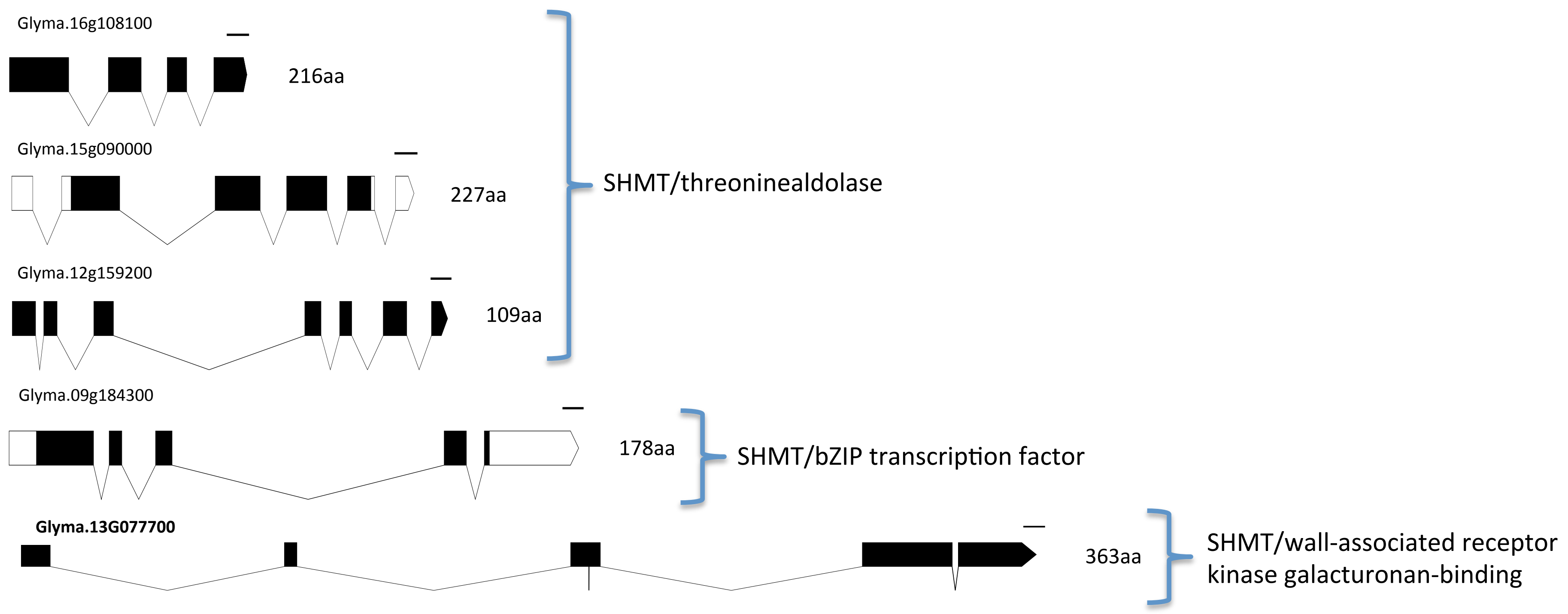

Supplementary Figure S2. Bifunctional Gm*SHMT* genomic DNA structure in soybean. Black boxes represent exons, lines represent introns. White boxes represent 5' UTR and 3' UTR untranslated regions. Five GmSHMTs with multiple-bifunctional activities including a SHMT/Threoninealdolase, SHMT/Receptor Kinase, SHMT/transcription factor activity all encoding short proteins. It is presumed that they has been diverged in their structure and function. Graphs were made using the Exon-Intron Graphic Maker Version 4, http://wormweb.org/exonintron. Scale represent 100 bp.

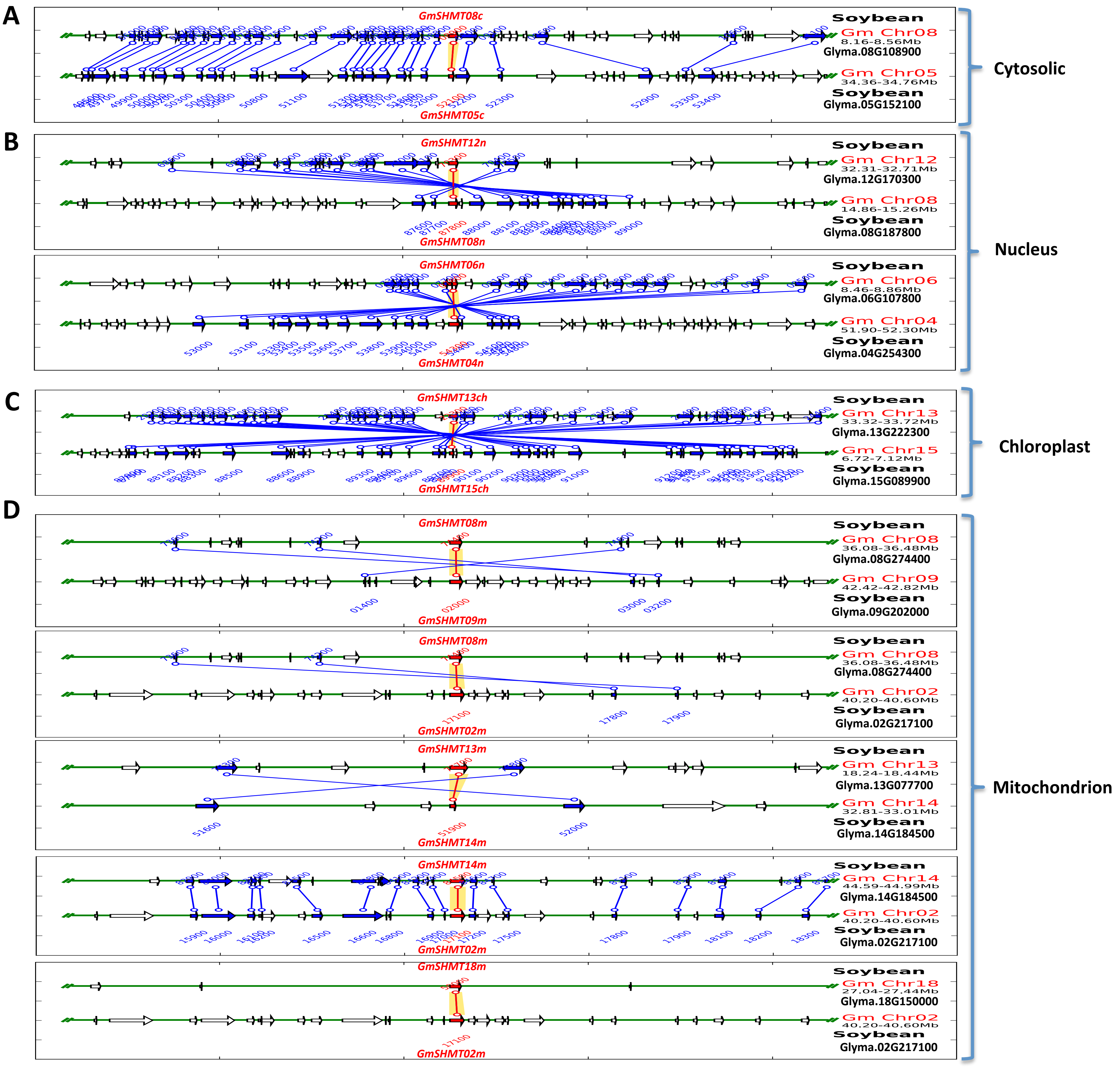

Supplementary Figure S3. Schematic representation of *GmSHMT* containing duplicated segments identified in the soybean genome. Soybean *SHMT* intragenome syntenic relationships were calculated using the Plant Genome Duplication Database. (**A**) Cytosolic *GmSHMTs* between chr08/chr05 belongs to a very large recent duplicated segment containing 551 additional conserved duplicated genes or anchors. (**B**) Nucleic *GmSHMTs* between chr12/chr08 and chr06/chr04 belong to another recent inverted duplicated segment containing 14 and 711 duplicated genes or anchors, respectively. (**C**) Chloroplastic GmSHMTs between chr13 and chr15 belongs to a fourth recent inverted duplicated segment containing 391 duplicated genes or anchors. (**D**) Mitochondrial *GmSHMT*s between chr08/chr09, chr08/chr02, and chr18/chr02 belongs to old duplicated segments containing 24, 10, and 7 duplicated genes or anchors. Graphs represent +/- 500 kb duplicated region centred in the *GmSHMT* genes.

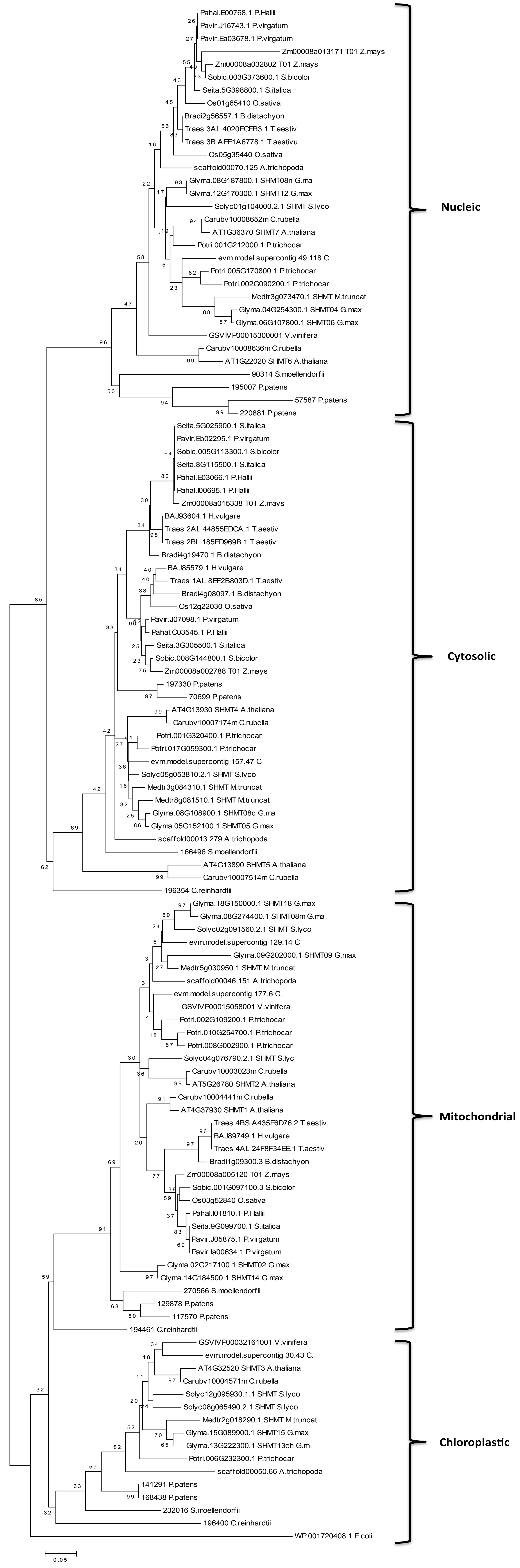

Supplementary Figure S4. Phylogenetic tree of SHMT from 22 plants species showing absence of chloroplastic-targeted SHMTs in monocots. All SHMT proteins identified in five model plants; C. reinhardtii (algae), P. patens (moss), S. moellendorfii (lycophyte), O. sativa (monocot), and A. thaliana (eudicot), in addition to G. max (soybean), in addition to other monocots and eudicots cytosolic, nucleic, chloroplastic, and mitochondrial-targeted SHMTs were included in the analysis. Extensive searches employing a considerable number of monocots including the plant model O. sativa, in addition to other monocots like Z. mays, S. bicolor, S. italica, H. vulgare, P. hallii, P. virgatum, and T. aestivum failed to identify any GmSHMT member within the chloroplastic clade. In addition, a remarkable decrease of chloroplastic-targeted GmSHMTs from all eudicots analysed was observed.

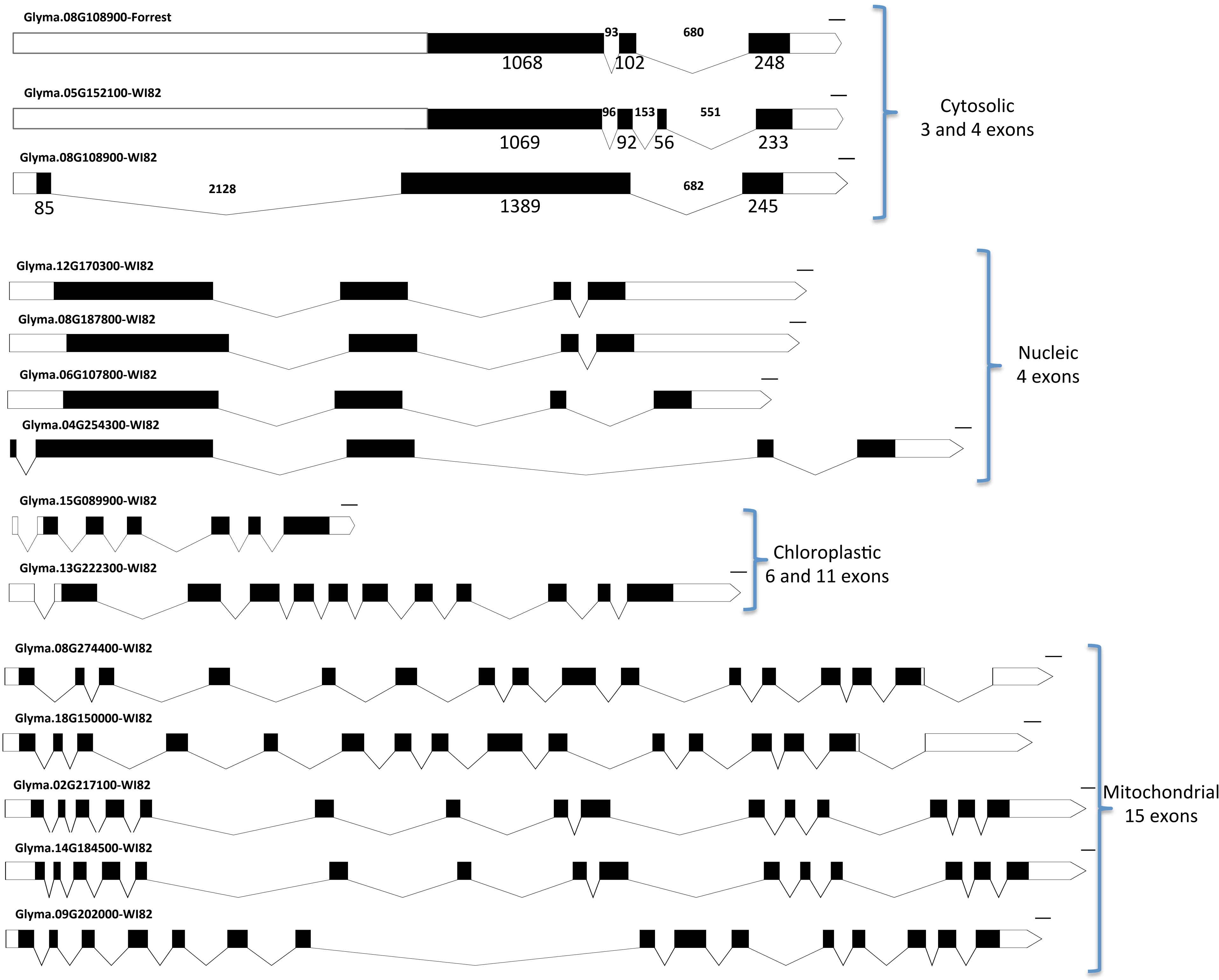

Supplementary Figure S5. *GmSHMT* genomic DNA structure in soybean. Black boxes represent exons, lines represent introns. White boxes represent 5' UTR and 3' UTR untranslated regions. Nucleic *GmSHMT*s has 4 Exons, mitochondrial *GmSHMT*s has 15 exons. Chroroplastic *GmSHMT13* has 11 exons, however the truncated *GmSHMT15* contains 6 exons only. Cytosolic *GmSHMT*s present different numbers of exons, whereas the *GmSHMT05* represent 4 exons, the *GmSHMT08* contained 3 exons only. Graphs were made using the Exon-Intron Graphic Maker Version 4, http://wormweb.org/exonintron. Scale represent 100 bp.

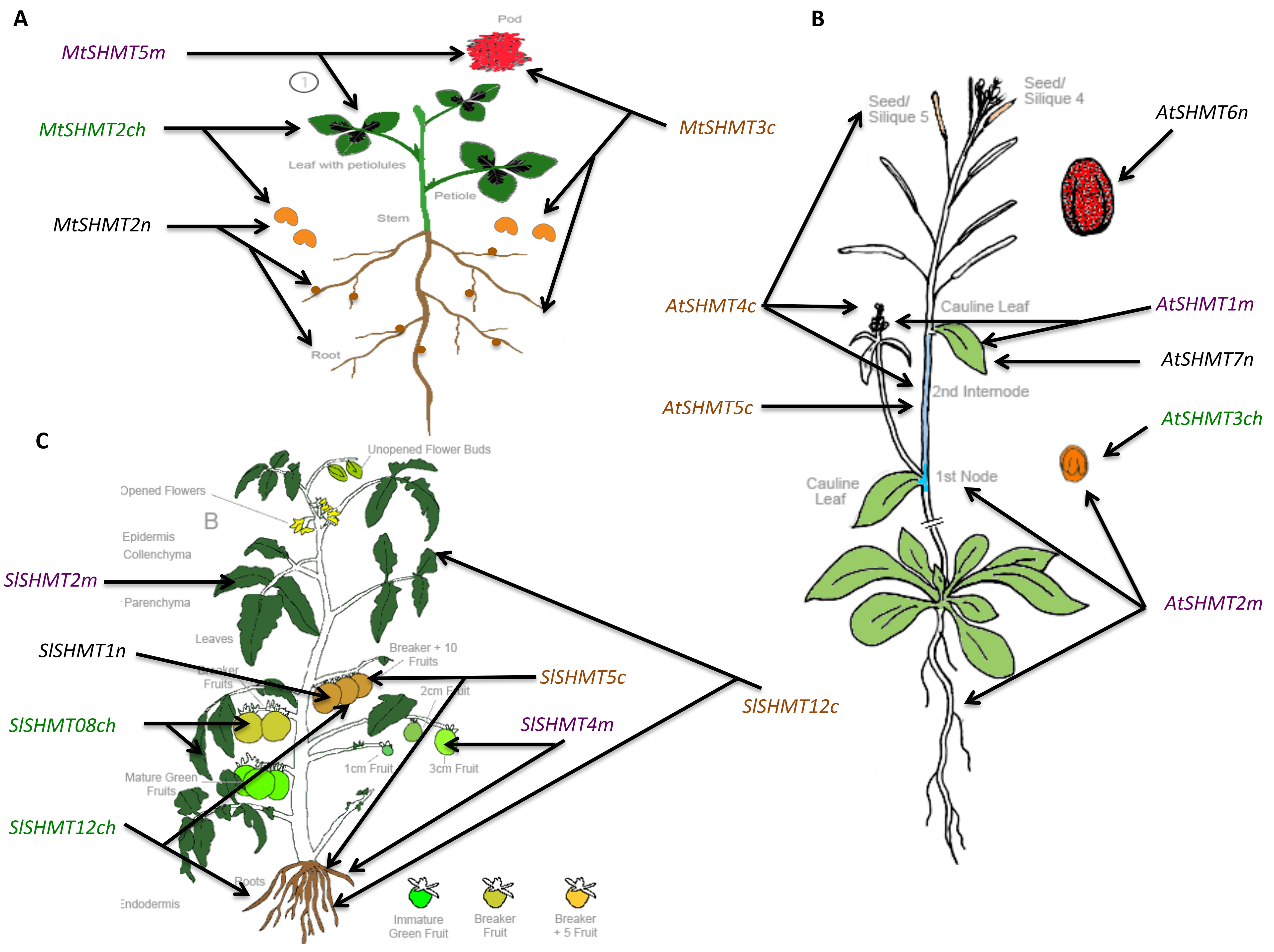

Supplementary Figure S6. Expression pattern of *Medicago truncatula* **(A)**, *Arabidopsis thaliana* **(B)**, and *Solanum lycopersicum* **(C)** *SHMT* gene members *in planta*, compiled from the public bio-analytic resource for plant biology database (http://bar.utoronto.ca) (Winter et al. 2007).

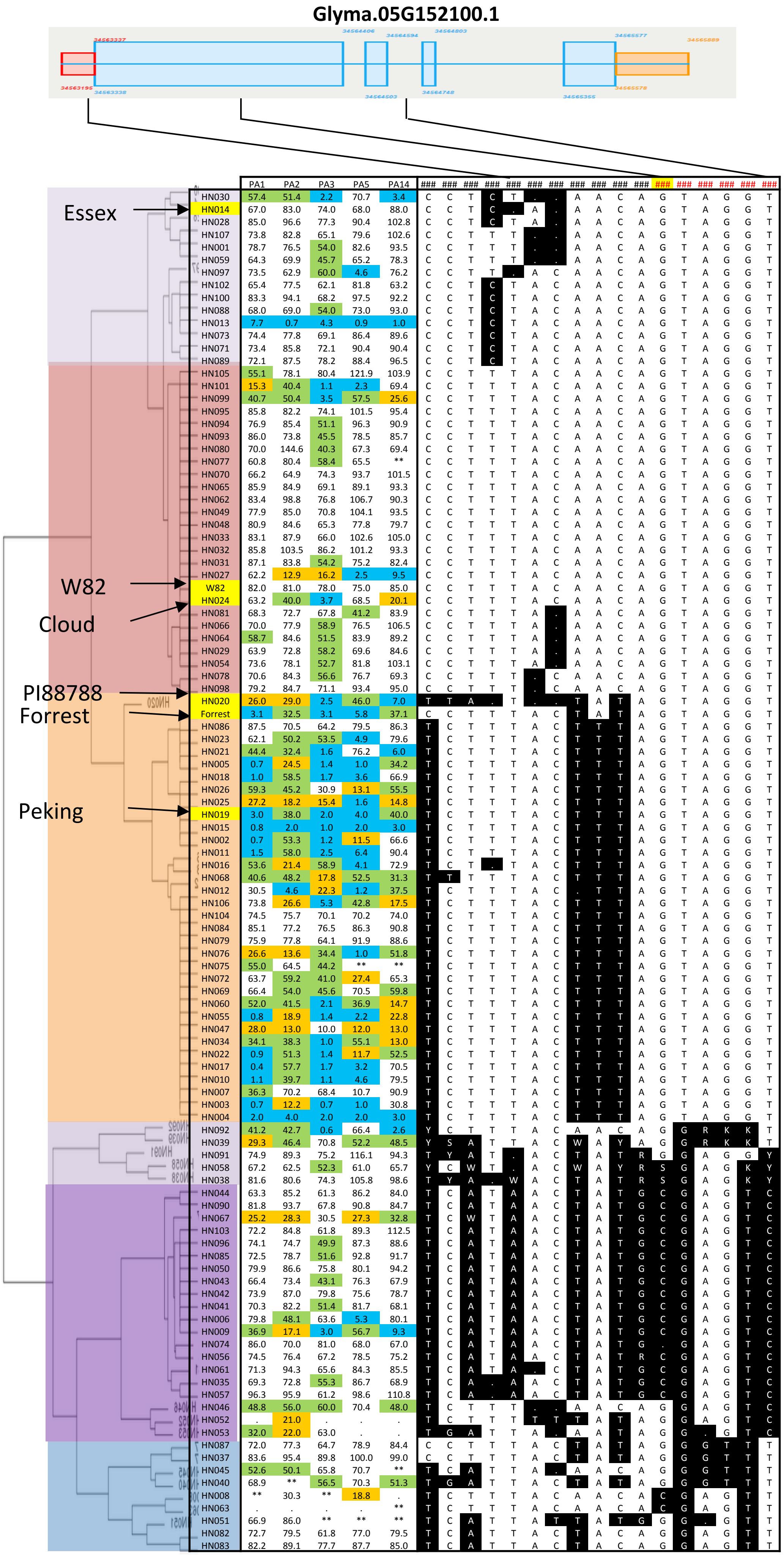

Supplementary Figure S7. **Haplotype clustering and correlation with soybean cyst nematode female index of the cytosolic-localized *GmSHMT05c* in the 106 soybean lines.** The 106 soybean lines included non-domesticated; semi-domesticated, and elite domesticated introductions belonging to the USDA soybean collection. Schematic graphs shows the position of SNP/indel for *Glyma.05g152100* (*GmSHMT05c*) gene. SNP in black background were specific to Peking-type of resistance, and clustered with soybean lines carrying resistance to three SCN Hg-types; 0, 2.7, and 2.5.7. Blue box represents exon, blue bar represents intron, orange box represents promoter region, grey box represents 3' or 5' UTR. SNPs were positioned relative to the genomic position in W82. SNP position in red text showing non-synonymous SNPs leading to amino acid change. Female Index in blue (FI<10, resistant to SCN), in green (10<FI<30, Moderate resistance), in orange (30<FI<60, Moderate susceptibility), in white (60<FI, Susceptible to SCN).

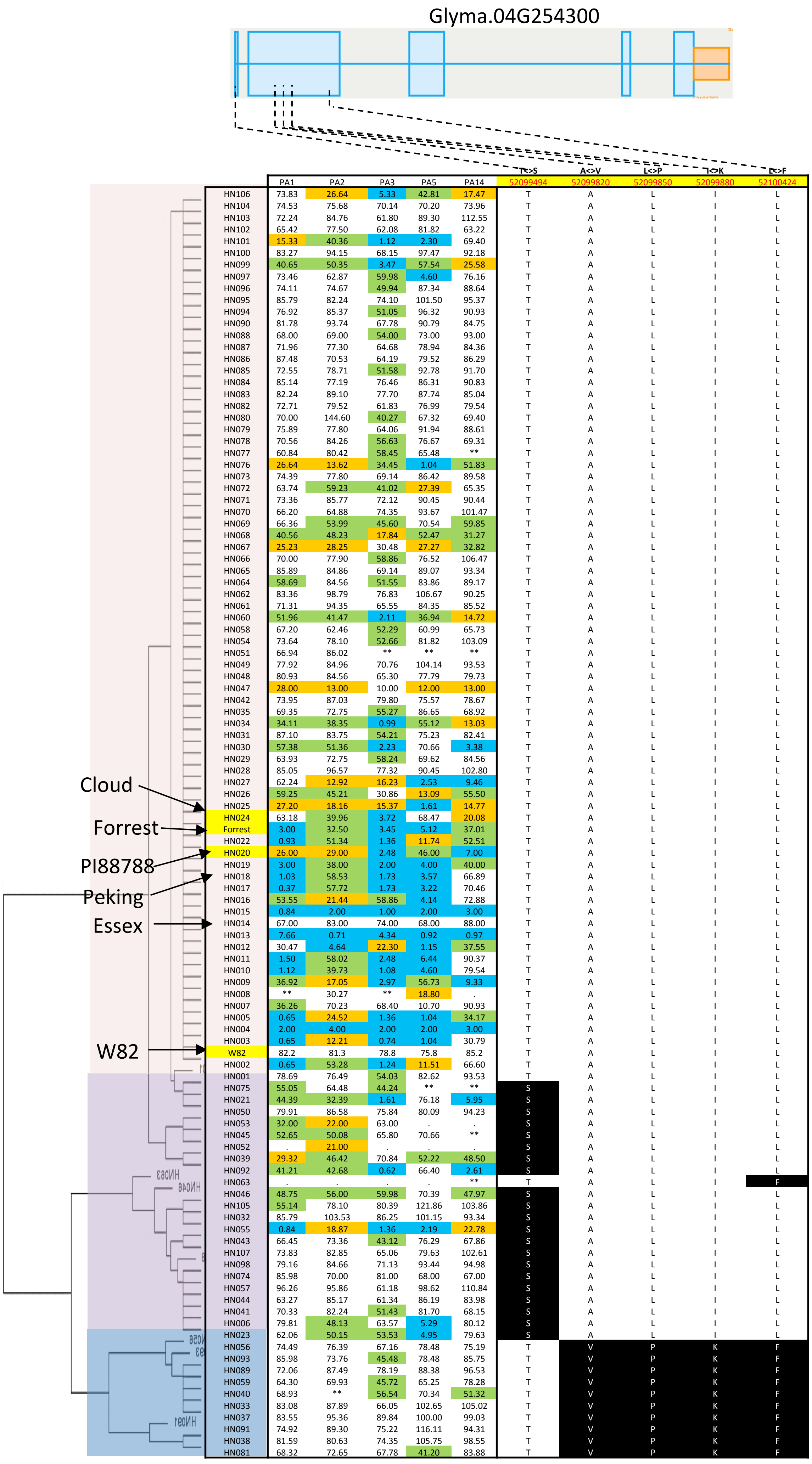

	PA1	PA2	PA3	PA5	PA14	T<>S 52099494	A<>V 52099820	L<>P 52099850	I<>K 52099880	L<>F 52100424
HN106	73.83	26.64	5.33	42.81	17.47	T	A	L	I	L
HN104	74.53	75.68	70.14	70.20	73.96	T	A	L	I	L
HN103	72.24	84.76	61.80	89.30	112.55	T	A	L	I	L
HN102	65.42	77.50	62.08	81.82	63.22	T	A	L	I	L
HN101	15.33	40.36	1.12	2.30	69.40	T	A	L	I	L
HN100	83.27	94.15	68.15	97.47	92.18	T	A	L	I	L
HN099	40.65	50.35	3.47	57.54	25.58	T	A	L	I	L
HN097	73.46	62.87	59.98	4.60	76.16	T	A	L	I	L
HN096	74.11	74.67	49.94	87.34	88.64	T	A	L	I	L
HN095	85.79	82.24	74.10	101.50	95.37	T	A	L	I	L
HN094	76.92	85.37	51.05	96.32	90.93	T	A	L	I	L
HN090	81.78	93.74	67.78	90.79	84.75	T	A	L	I	L
HN088	68.00	69.00	54.00	73.00	93.00	T	A	L	I	L
HN087	71.96	77.30	64.68	78.94	84.36	T	A	L	I	L
HN086	87.48	70.53	64.19	79.52	86.29	T	A	L	I	L
HN085	72.55	78.71	51.58	92.78	91.70	T	A	L	I	L
HN084	85.14	77.19	76.46	86.31	90.83	T	A	L	I	L
HN083	82.24	89.10	77.70	87.74	85.04	T	A	L	I	L
HN082	72.71	79.52	61.83	76.99	79.54	T	A	L	I	L
HN080	70.00	144.60	40.27	67.32	69.40	T	A	L	I	L
HN079	75.89	77.80	64.06	91.94	88.61	T	A	L	I	L
HN078	70.56	84.26	56.63	76.67	69.31	T	A	L	I	L
HN077	60.84	80.42	58.45	65.48	**	T	A	L	I	L
HN076	26.64	13.62	34.45	1.04	51.83	T	A	L	I	L
HN073	74.39	77.80	69.14	86.42	89.58	T	A	L	I	L
HN072	63.74	59.23	41.02	27.39	65.35	T	A	L	I	L
HN071	73.36	85.77	72.12	90.45	90.44	T	A	L	I	L
HN070	66.20	64.88	74.35	93.67	101.47	T	A	L	I	L
HN069	66.36	53.99	45.60	70.54	59.85	T	A	L	I	L
HN068	40.56	48.23	17.84	52.47	31.27	T	A	L	I	L
HN067	25.23	28.25	30.48	27.27	32.82	T	A	L	I	L
HN066	70.00	77.90	58.86	76.52	106.47	T	A	L	I	L
HN065	85.89	84.86	69.14	89.07	93.34	T	A	L	I	L
HN064	58.69	84.56	51.55	83.86	89.17	T	A	L	I	L
HN062	83.36	98.79	76.83	106.67	90.25	T	A	L	I	L
HN061	71.31	94.35	65.55	84.35	85.52	T	A	L	I	L
HN060	51.96	41.47	2.11	36.94	14.72	T	A	L	I	L
HN058	67.20	62.46	52.29	60.99	65.73	T	A	L	I	L
HN054	73.64	78.10	52.66	81.82	103.09	T	A	L	I	L
HN051	66.94	86.02	**	**	**	T	A	L	I	L
HN049	77.92	84.96	70.76	104.14	93.53	T	A	L	I	L
HN048	80.93	84.56	65.30	77.79	79.73	T	A	L	I	L
HN047	28.00	13.00	10.00	12.00	13.00	T	A	L	I	L
HN042	73.95	87.03	79.80	75.57	78.67	T	A	L	I	L
HN035	69.35	72.75	55.27	86.65	68.92	T	A	L	I	L
HN034	34.11	38.35	0.99	55.12	13.03	T	A	L	I	L
HN031	87.10	83.75	54.21	75.23	82.41	T	A	L	I	L
HN030	57.38	51.36	2.23	70.66	3.38	T	A	L	I	L
HN029	63.93	72.75	58.24	69.62	84.56	T	A	L	I	L
HN028	85.05	96.57	77.32	90.45	102.80	T	A	L	I	L
HN027	62.24	12.92	16.23	2.53	9.46	T	A	L	I	L
HN026	59.25	45.21	30.86	13.09	55.50	T	A	L	I	L
HN025	27.20	18.16	15.37	1.61	14.77	T	A	L	I	L
HN024	63.18	39.96	3.72	68.47	20.08	T	A	L	I	L
Forrest	3.00	32.50	3.45	5.12	37.01	T	A	L	I	L
HN022	0.93	51.34	1.36	11.74	52.51	T	A	L	I	L
HN020	26.00	29.00	2.48	46.00	7.00	T	A	L	I	L
HN019	3.00	38.00	2.00	4.00	40.00	T	A	L	I	L
HN018	1.03	58.53	1.73	3.57	66.89	T	A	L	I	L
HN017	0.37	57.72	1.73	3.22	70.46	T	A	L	I	L
HN016	53.55	21.44	58.86	4.14	72.88	T	A	L	I	L
HN015	0.84	2.00	1.00	2.00	3.00	T	A	L	I	L
HN014	67.00	83.00	74.00	68.00	88.00	T	A	L	I	L
HN013	7.66	0.71	4.34	0.92	0.97	T	A	L	I	L
HN012	30.47	4.64	22.30	1.15	37.55	T	A	L	I	L
HN011	1.50	58.02	2.48	6.44	90.37	T	A	L	I	L
HN010	1.12	39.73	1.08	4.60	79.54	T	A	L	I	L
HN009	36.92	17.05	2.97	56.73	9.33	T	A	L	I	L
HN008	**	30.27	**	18.80	.	T	A	L	I	L
HN007	36.26	70.23	68.40	10.70	90.93	T	A	L	I	L
HN005	0.65	24.52	1.36	1.04	34.17	T	A	L	I	L
HN004	2.00	4.00	2.00	2.00	3.00	T	A	L	I	L
HN003	0.65	12.21	0.74	1.04	30.79	T	A	L	I	L
W82	82.2	81.3	78.8	75.8	85.2	T	A	L	I	L
HN002	0.65	53.28	1.24	11.51	66.60	T	A	L	I	L
HN001	78.69	76.49	54.03	82.62	93.53	T	A	L	I	L
HN075	55.05	64.48	44.24	**	**	S	A	L	I	L
HN021	44.39	32.39	1.61	76.18	5.95	S	A	L	I	L
HN050	79.91	86.58	75.84	80.09	94.23	S	A	L	I	L
HN053	32.00	22.00	63.00	.	.	S	A	L	I	L
HN045	52.65	50.08	65.80	70.66	**	S	A	L	I	L
HN052	.	21.00	.	.	.	S	A	L	I	L
HN039	29.32	46.42	70.84	52.22	48.50	S	A	L	I	L
HN092	41.21	42.68	0.62	66.40	2.61	S	A	L	I	L
HN063	.	.	.	.	**	T	A	L	I	F
HN046	48.75	56.00	59.98	70.39	47.97	S	A	L	I	L
HN105	55.14	78.10	80.39	121.86	103.86	S	A	L	I	L
HN032	85.79	103.53	86.25	101.15	93.34	S	A	L	I	L
HN055	0.84	18.87	1.36	2.19	22.78	S	A	L	I	L
HN043	66.45	73.36	43.12	76.29	67.86	S	A	L	I	L
HN107	73.83	82.85	65.06	79.63	102.61	S	A	L	I	L
HN098	79.16	84.66	71.13	93.44	94.98	S	A	L	I	L
HN074	85.98	70.00	81.00	68.00	67.00	S	A	L	I	L
HN057	96.26	95.86	61.18	98.62	110.84	S	A	L	I	L
HN044	63.27	85.17	61.34	86.19	83.98	S	A	L	I	L
HN041	70.33	82.24	51.43	81.70	68.15	S	A	L	I	L
HN006	79.81	48.13	63.57	5.29	80.12	S	A	L	I	L
HN023	62.06	50.15	53.53	4.95	79.63	S	A	L	I	L
HN056	74.49	76.39	67.16	78.48	75.19	T	V	P	K	F
HN093	85.98	73.76	45.48	78.48	85.75	T	V	P	K	F
HN089	72.06	87.49	78.19	88.38	96.53	T	V	P	K	F
HN059	64.30	69.93	45.72	65.25	78.28	T	V	P	K	F
HN040	68.93	**	56.54	70.34	51.32	T	V	P	K	F
HN033	83.08	87.89	66.05	102.65	105.02	T	V	P	K	F
HN037	83.55	95.36	89.84	100.00	99.03	T	V	P	K	F
HN091	74.92	89.30	75.22	116.11	94.31	T	V	P	K	F
HN038	81.59	80.63	74.35	105.75	98.55	T	V	P	K	F
HN081	68.32	72.65	67.78	41.20	83.88	T	V	P	K	F

Supplementary Figure S8. **Haplotype clustering and correlation with soybean cyst nematode female index of the nucleic-localized *GmSHMT04n* in the 106 soybean lines.** The 106 soybean lines included non-domesticated; semi-domesticated, and elite domesticated introductions belonging to the USDA soybean collection. Schematic graphs shows the position of SNP/indel for *Glyma.04g254300* (*GmSHMT04n*) gene. SNP in black background were specific to Peking-type of resistance, and clustered with soybean lines carrying resistance to three SCN Hg-types; 0, 2.7, and 2.5.7. Blue box represents exon, blue bar represents intron, orange box represents promoter region, grey box represents 3' or 5' UTR. SNPs were positioned relative to the genomic position in W82. SNP position in red text showing non-synonymous SNPs leading to amino acid change. Female Index in blue (FI<10, resistant to SCN), in green (10<FI<30, Moderate resistance), in orange (30<FI<60, Moderate susceptibility), in white (60<FI, Susceptible to SCN).

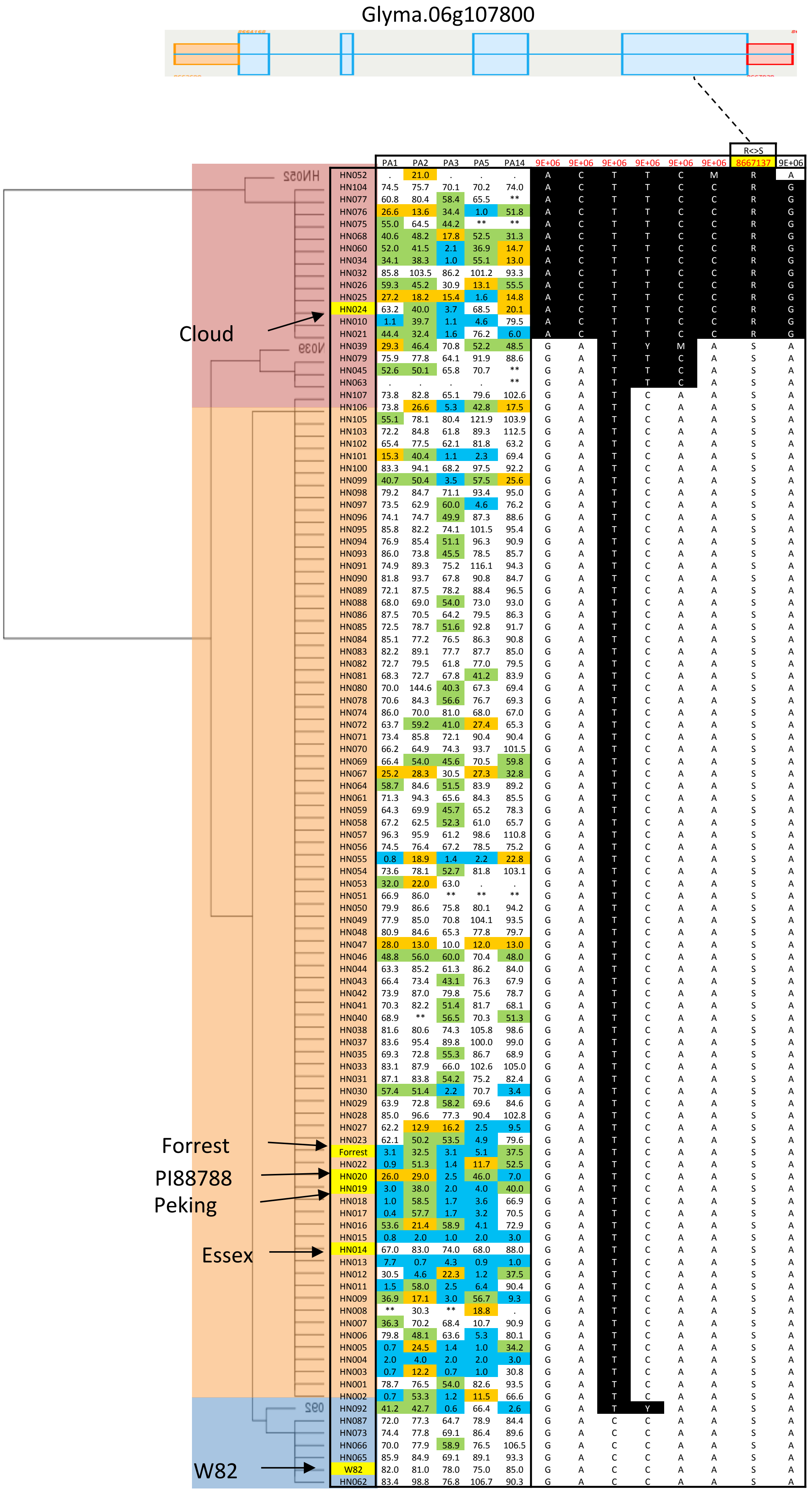

Supplementary Figure S9. **Haplotype clustering and correlation with soybean cyst nematode female index of the nucleic-localized *GmSHMT06n* in the 106 soybean lines.** The 106 soybean lines included non-domesticated; semi-domesticated, and elite domesticated introductions belonging to the USDA soybean collection. Schematic graphs shows the position of SNP/indel for *Glyma.06g107800* (*GmSHMT06n*) gene. SNP in black background were specific to Peking-type of resistance, and clustered with soybean lines carrying resistance to three SCN Hg-types; 0, 2.7, and 2.5.7. Blue box represents exon, blue bar represents intron, orange box represents promoter region, grey box represents 3' or 5' UTR. SNPs were positioned relative to the genomic position in W82. SNP position in red text showing non-synonymous SNPs leading to amino acid change. Female Index in blue (FI<10, resistant to SCN), in green (10<FI<30, Moderate resistance), in orange (30<FI<60, Moderate susceptibility), in white (60<FI, Susceptible to SCN).

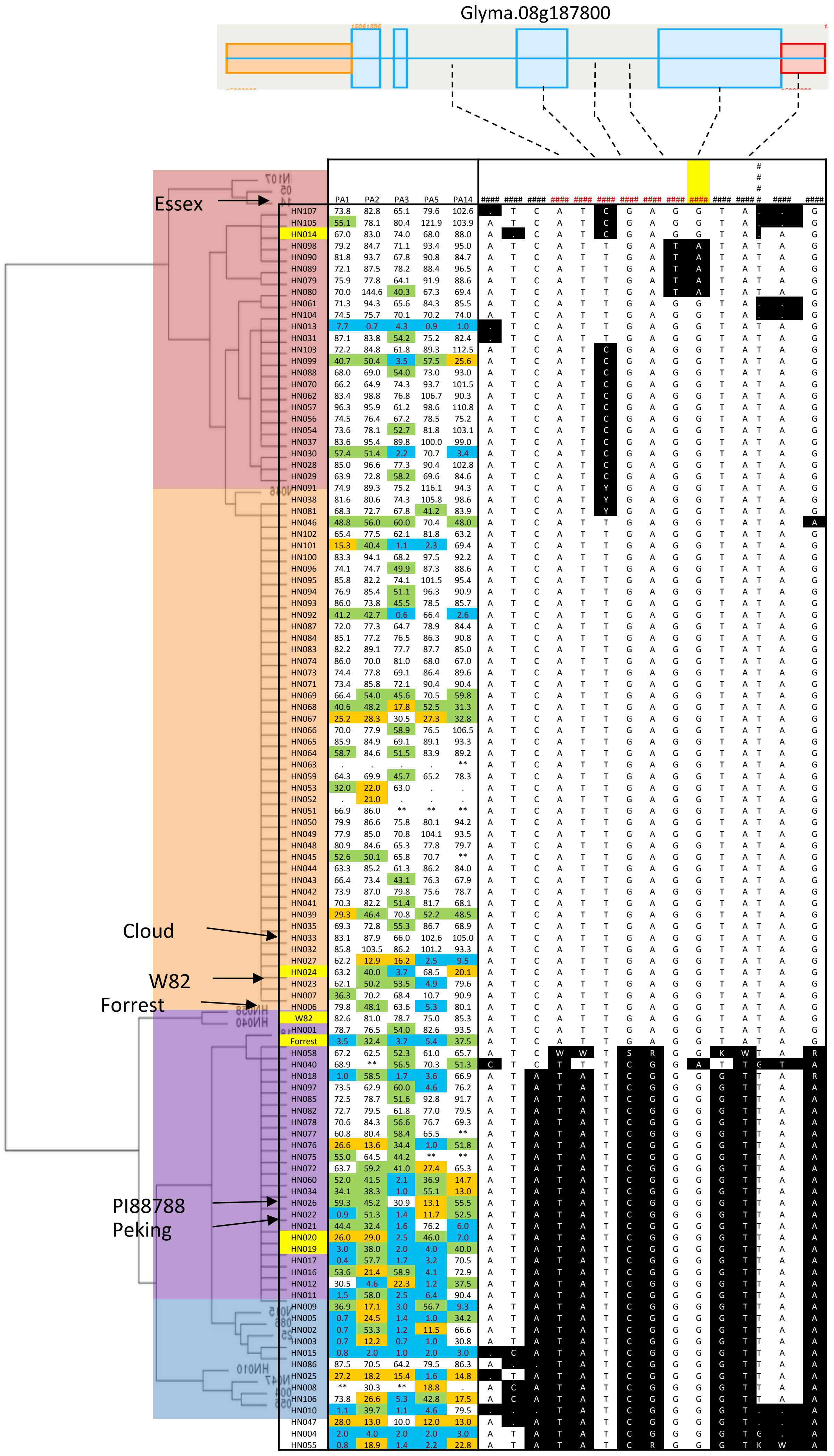

Supplementary Figure S10. **Haplotype clustering and correlation with soybean cyst nematode female index of the nucleic-localized *GmSHMT08n* in the 106 soybean lines.** The 106 soybean lines included non-domesticated; semi-domesticated, and elite domesticated introductions belonging to the USDA soybean collection. Schematic graphs shows the position of SNP/indel for *Glyma.08g187800* (*GmSHMT08n*) gene. SNP in black background were specific to Peking-type of resistance, and clustered with soybean lines carrying resistance to three SCN Hg-types; 0, 2.7, and 2.5.7. Blue box represents exon, blue bar represents intron, orange box represents promoter region, grey box represents 3' or 5' UTR. SNPs were positioned relative to the genomic position in W82. SNP position in red text showing non-synonymous SNPs leading to amino acid change. Female Index in blue (FI<10, resistant to SCN), in green (10<FI<30, Moderate resistance), in orange (30<FI<60, Moderate susceptibility), in white (60<FI, Susceptible to SCN).

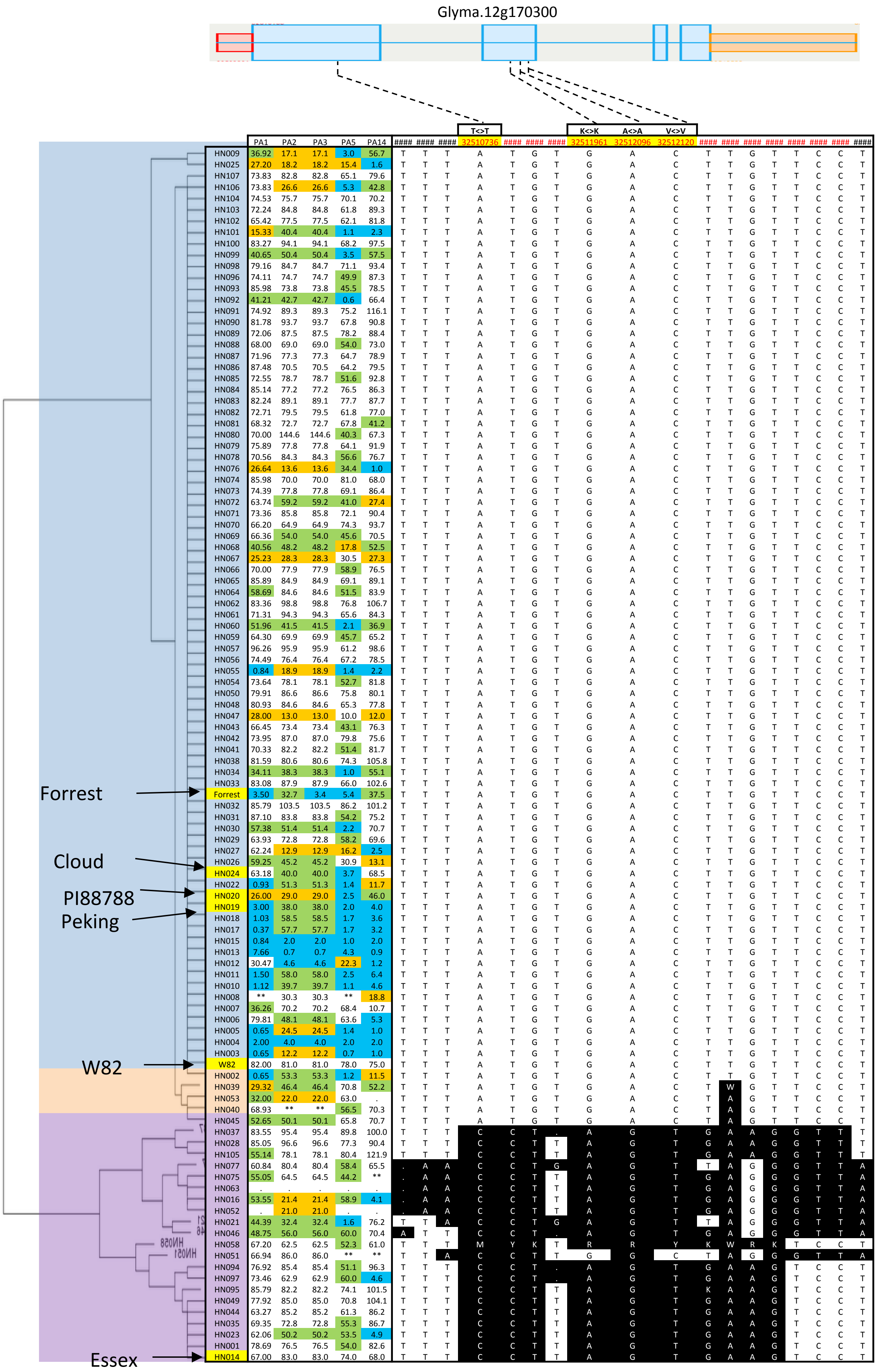

Supplementary Figure S11. **Haplotype clustering and correlation with soybean cyst nematode female index of the nucleic-localized *GmSHMT12n* in the 106 soybean lines.** The 106 soybean lines included non-domesticated; semi-domesticated, and elite domesticated introductions belonging to the USDA soybean collection. Schematic graphs shows the position of SNP/indel for *Glyma.12g170300* (*GmSHMT12n*) gene. SNP in black background were specific to Peking-type of resistance, and clustered with soybean lines carrying resistance to three SCN Hg-types; 0, 2.7, and 2.5.7. Blue box represents exon, blue bar represents intron, orange box represents promoter region, grey box represents 3' or 5' UTR. SNPs were positioned relative to the genomic position in W82. SNP position in red text showing non-synonymous SNPs leading to amino acid change. Female Index in blue (FI<10, resistant to SCN), in green (10<FI<30, Moderate resistance), in orange (30<FI<60, Moderate susceptibility), in white (60<FI, Susceptible to SCN).

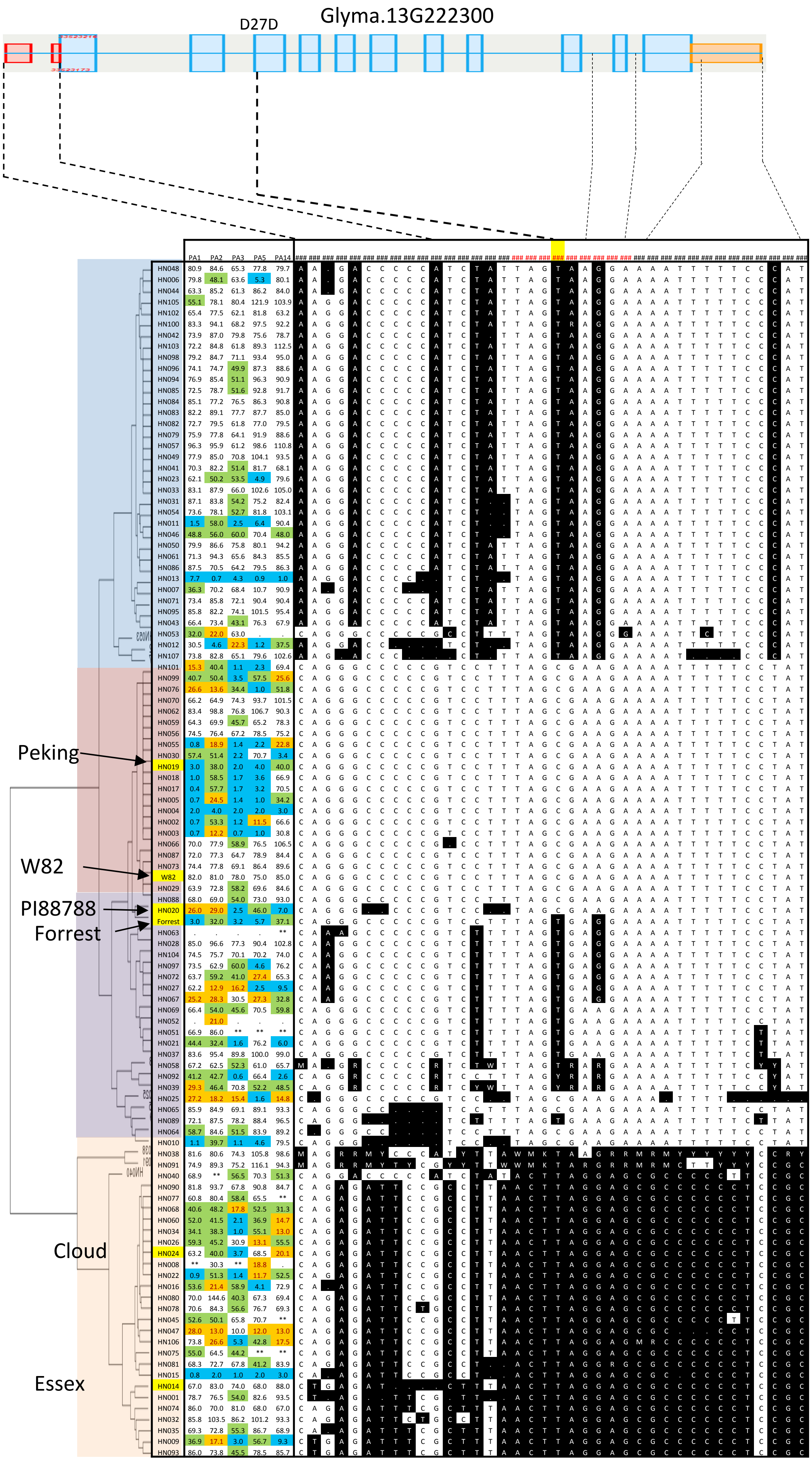

Supplementary Figure S12. **Haplotype clustering and correlation with soybean cyst nematode female index of the chloroplastic-localized *GmSHMT13ch* in the 106 soybean lines.** The 106 soybean lines included non-domesticated; semi-domesticated, and elite domesticated introductions belonging to the USDA soybean collection. Schematic graphs shows the position of SNP/indel for *Glyma.13g222300* (*GmSHMT13ch*) gene. SNP in black background were specific to Peking-type of resistance, and clustered with soybean lines carrying resistance to three SCN Hg-types; 0, 2.7, and 2.5.7. Blue box represents exon, blue bar represents intron, orange box represents promoter region, grey box represents 3' or 5' UTR. SNPs were positioned relative to the genomic position in W82. SNP position in red text showing non-synonymous SNPs leading to amino acid change. Female Index in blue (FI<10, resistant to SCN), in green (10<FI<30, Moderate resistance), in orange (30<FI<60, Moderate susceptibility), in white (60<FI, Susceptible to SCN).

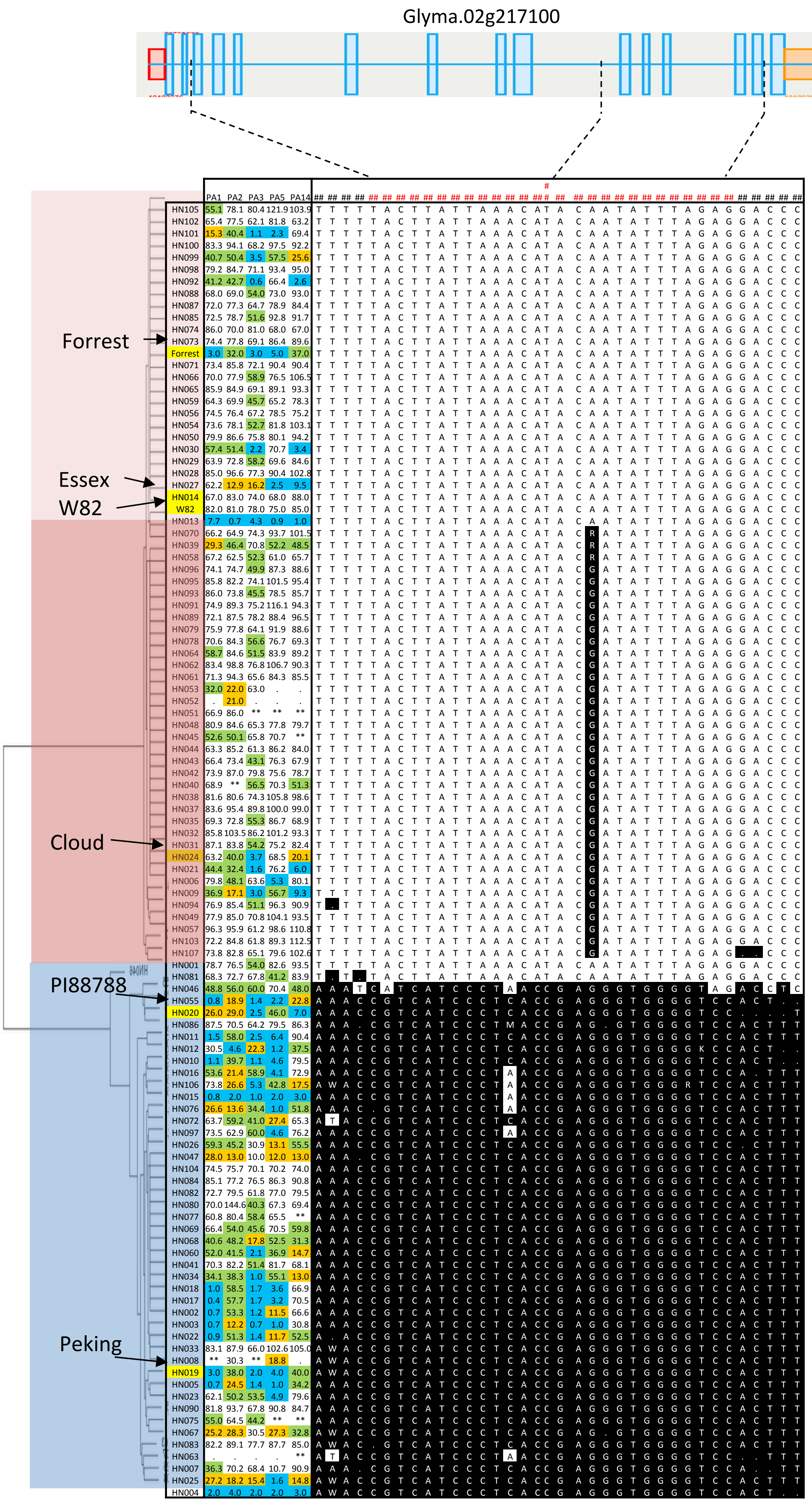

Supplementary Figure S13. **Haplotype clustering and correlation with soybean cyst nematode female index of the mitochondrial-localized *GmSHMT02m* in the 106 soybean lines.** The 106 soybean lines included non-domesticated; semi-domesticated, and elite domesticated introductions belonging to the USDA soybean collection. Schematic graphs shows the position of SNP/indel for *Glyma.02g217100* (*GmSHMT02m*) gene. SNP in black background were specific to Peking-type of resistance, and clustered with soybean lines carrying resistance to three SCN Hg-types; 0, 2.7, and 2.5.7. Blue box represents exon, blue bar represents intron, orange box represents promoter region, grey box represents 3' or 5' UTR. SNPs were positioned relative to the genomic position in W82. SNP position in red text showing non-synonymous SNPs leading to amino acid change. Female Index in blue (FI<10, resistant to SCN), in green (10<FI<30, Moderate resistance), in orange (30<FI<60, Moderate susceptibility), in white (60<FI, Susceptible to SCN).

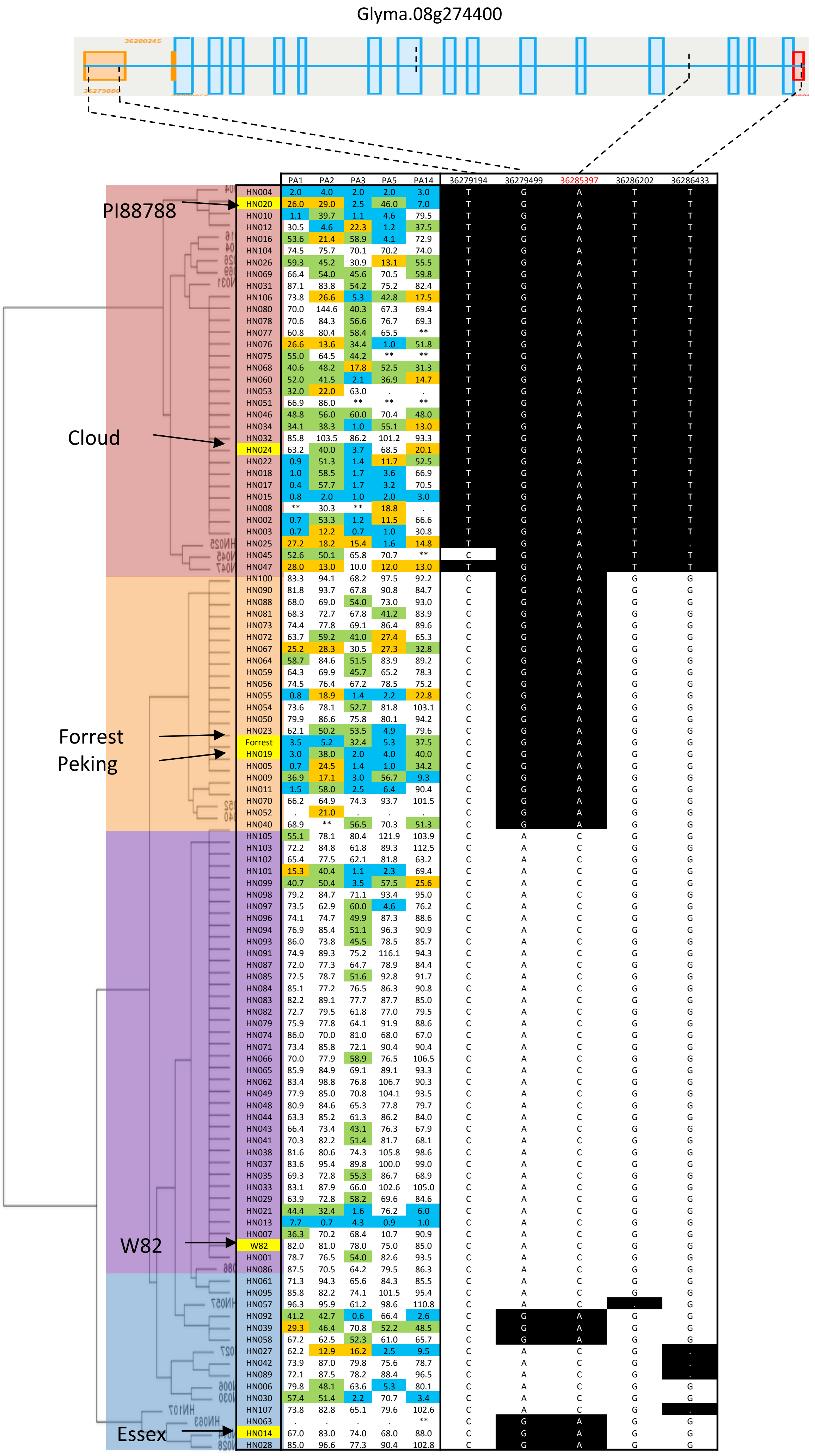

Supplementary Figure S14. **Haplotype clustering and correlation with soybean cyst nematode female index of the mitochondrial-localized *GmSHMT08m* in the 106 soybean lines.** The 106 soybean lines included non-domesticated; semi-domesticated, and elite domesticated introductions belonging to the USDA soybean collection. Schematic graphs shows the position of SNP/indel for *Glyma.08g274400* (*GmSHMT08m*) gene. SNP in black background were specific to Peking-type of resistance, and clustered with soybean lines carrying resistance to three SCN Hg-types; 0, 2.7, and 2.5.7. Blue box represents exon, blue bar represents intron, orange box represents promoter region, grey box represents 3' or 5' UTR. SNPs were positioned relative to the genomic position in W82. SNP position in red text showing non-synonymous SNPs leading to amino acid change. Female Index in blue (FI<10, resistant to SCN), in green (10<FI<30, Moderate resistance), in orange (30<FI<60, Moderate susceptibility), in white (60<FI, Susceptible to SCN).

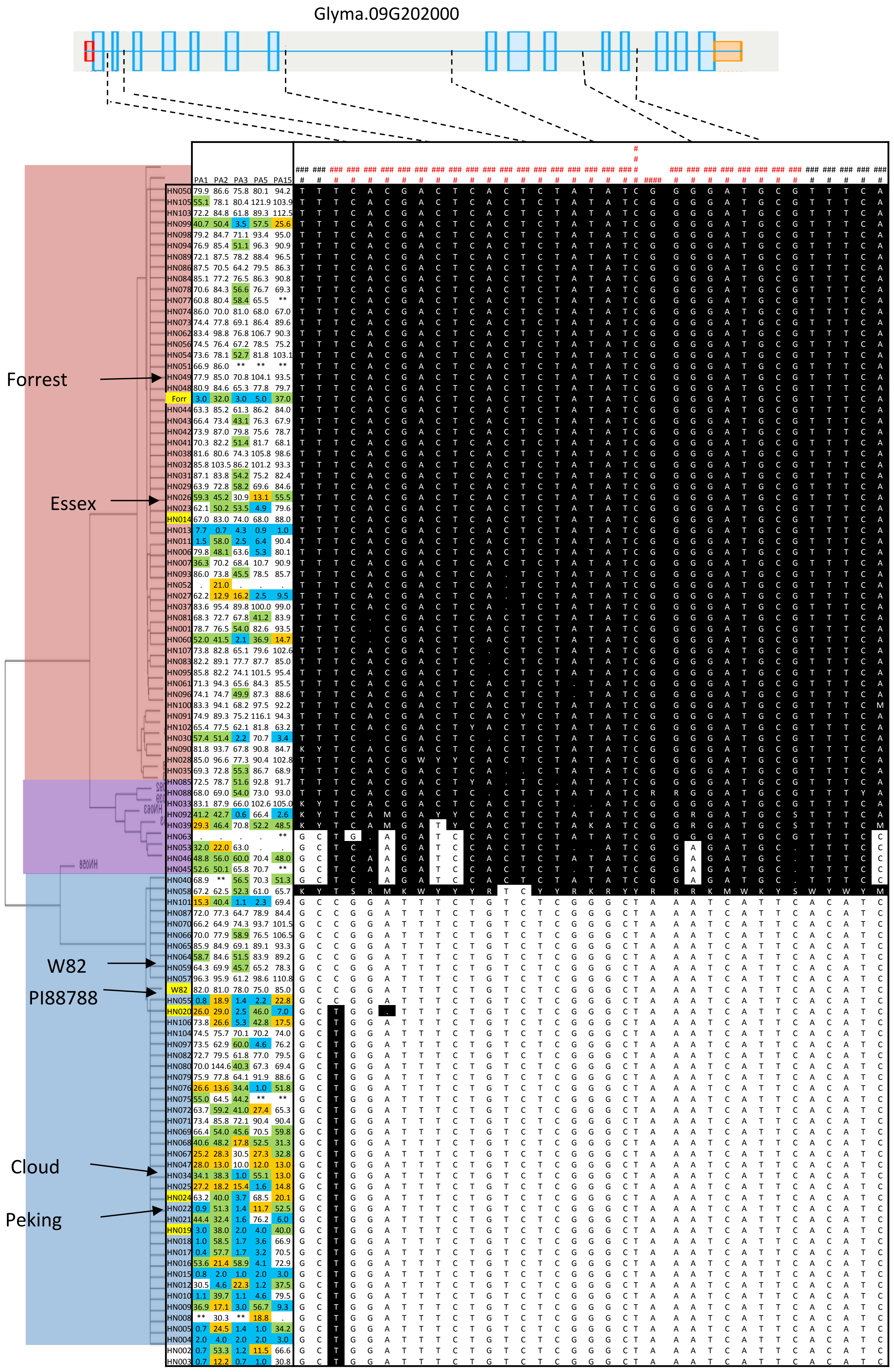

Supplementary Figure S15. Haplotype clustering and correlation with soybean cyst nematode female index of the mitochondrial-localized *GmSHMT09m* in the 106 soybean lines. The 106 soybean lines included non-domesticated; semi-domesticated, and elite domesticated introductions belonging to the USDA soybean collection. Schematic graphs shows the position of SNP/indel for *Glyma.09g217100* (*GmSHMT09m*) gene. SNP in black background were specific to Peking-type of resistance, and clustered with soybean lines carrying resistance to three SCN Hg-types; 0, 2.7, and 2.5.7. Blue box represents exon, blue bar represents intron, orange box represents promoter region, grey box represents 3' or 5' UTR. SNPs were positioned relative to the genomic position in W82. SNP position in red text showing non-synonymous SNPs leading to amino acid change. Female Index in blue (FI<10, resistant to SCN), in green (10<FI<30, Moderate resistance), in orange (30<FI<60, Moderate susceptibility), in white (60<FI, Susceptible to SCN).

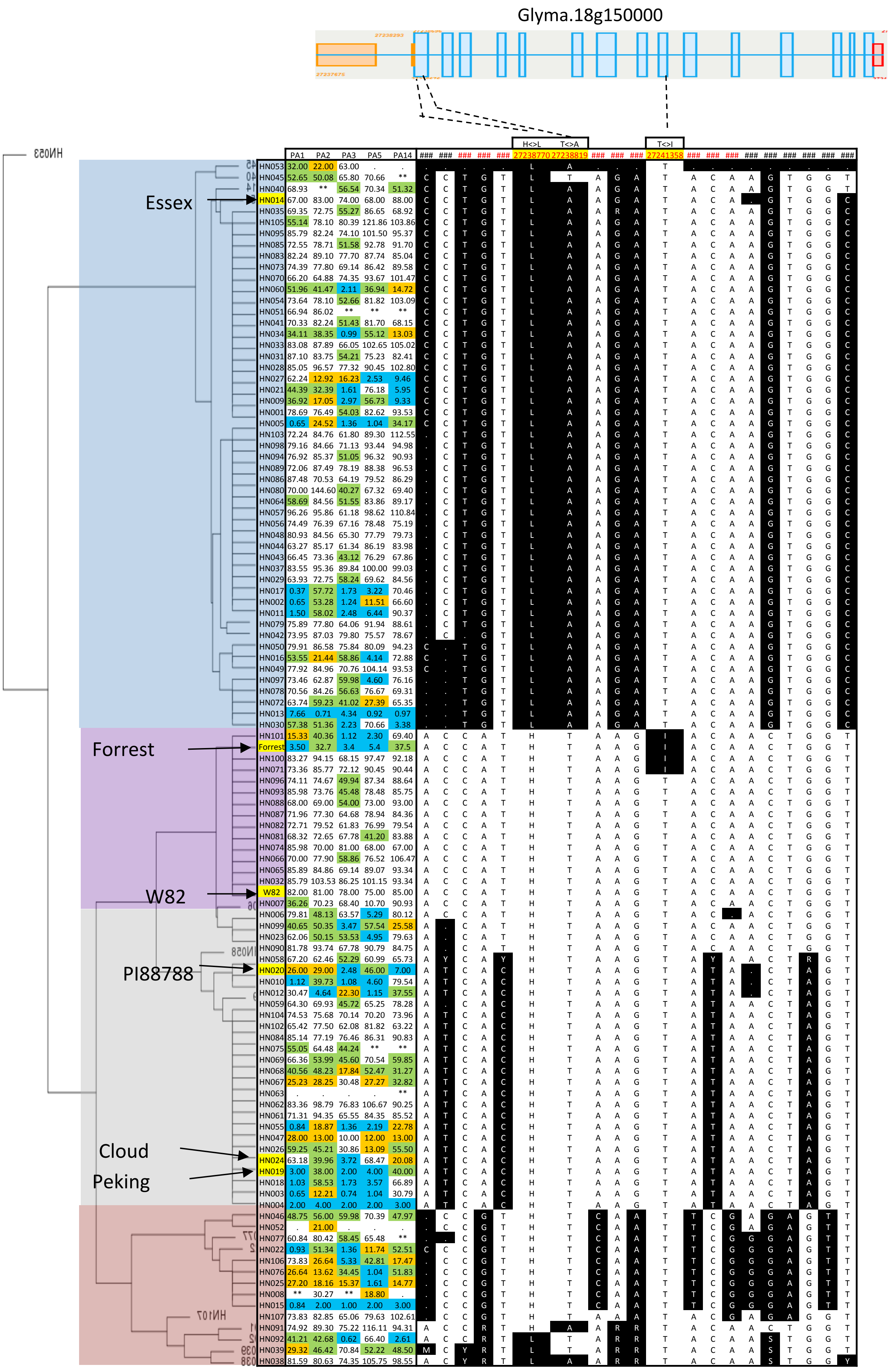

Supplementary Figure S16. **Haplotype clustering and correlation with soybean cyst nematode female index of the mitochondrial-localized *GmSHMT18m* in the 106 soybean lines.** The 106 soybean lines included non-domesticated; semi-domesticated, and elite domesticated introductions belonging to the USDA soybean collection. Schematic graphs shows the position of SNP/indel for *Glyma.18g150000* (*GmSHMT18m*) gene. SNP in black background were specific to Peking-type of resistance, and clustered with soybean lines carrying resistance to three SCN Hg-types; 0, 2.7, and 2.5.7. Blue box represents exon, blue bar represents intron, orange box represents promoter region, grey box represents 3' or 5' UTR. SNPs were positioned relative to the genomic position in W82. SNP position in red text showing non-synonymous SNPs leading to amino acid change. Female Index in blue (FI<10, resistant to SCN), in green (10<FI<30, Moderate resistance), in orange (30<FI<60, Moderate susceptibility), in white (60<FI, Susceptible to SCN).

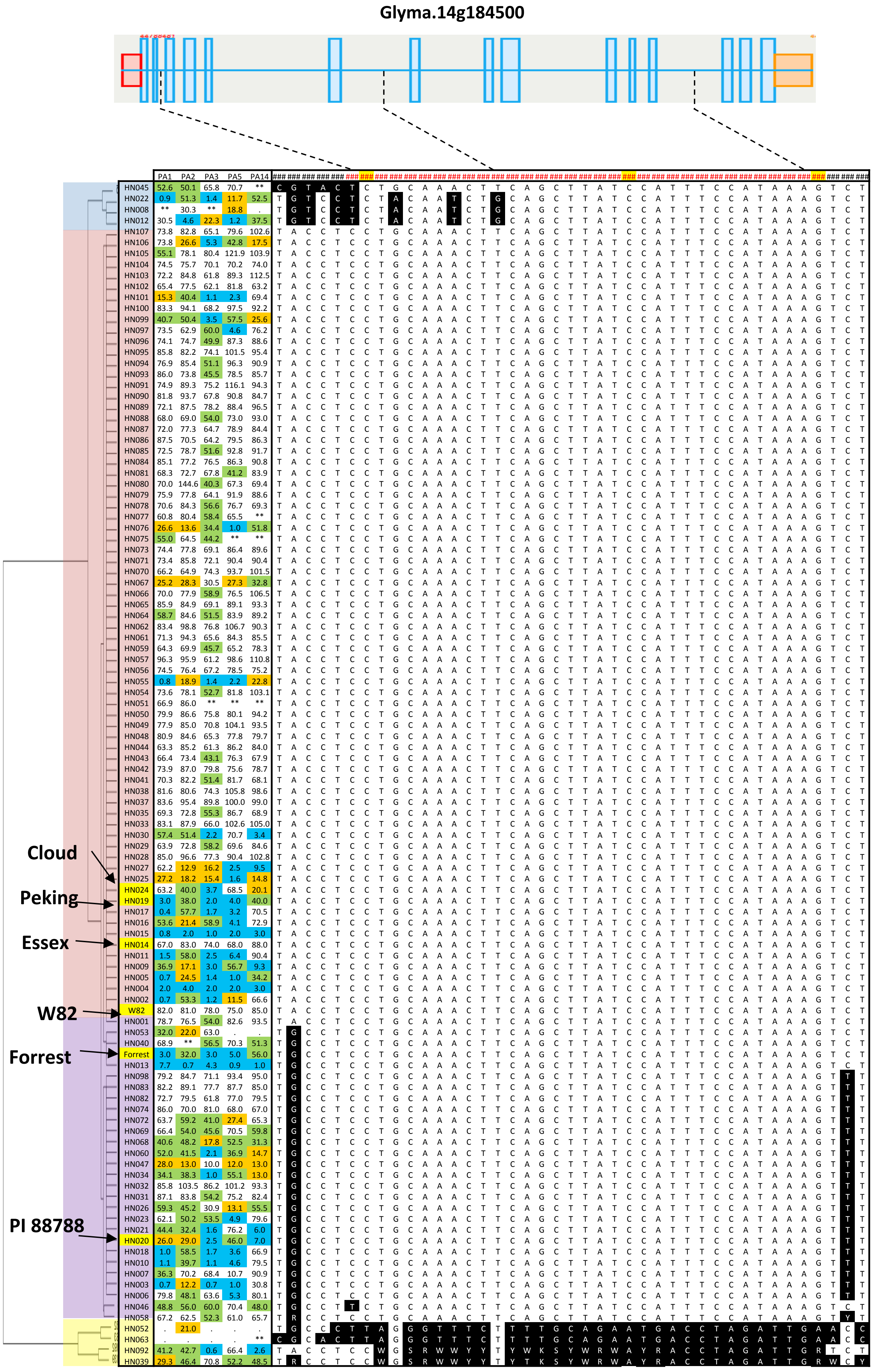

Supplementary Figure S17. **Haplotype clustering and correlation with soybean cyst nematode female index of the mitochondrial-localized *GmSHMT14m* in the 106 soybean lines.** The 106 soybean lines included non-domesticated; semi-domesticated, and elite domesticated introductions belonging to the USDA soybean collection. Schematic graphs shows the position of SNP/indel for *Glyma.14g184500* (*GmSHMT14m*) gene. SNP in black background were specific to Peking-type of resistance, and clustered with soybean lines carrying resistance to three SCN Hg-types; 0, 2.7, and 2.5.7. Blue box represents exon, blue bar represents intron, orange box represents promoter region, grey box represents 3' or 5' UTR. SNPs were positioned relative to the genomic position in W82. SNP position in red text showing non-synonymous SNPs leading to amino acid change. Female Index in blue (FI<10, resistant to SCN), in green (10<FI<30, Moderate resistance), in orange (30<FI<60, Moderate susceptibility), in white (60<FI, Susceptible to SCN).

Supplemental Table S1. *GmSHMT*s intragenome syntenic relationship calculations showing independent duplicate blocks containing the genomic pairs of *GmSHMT* family members with their corresponding number of conserved genes or anchors. Soybean genome duplicated chromosomal segments containing *GmSHMT*s were calculated using the Plant Genome Duplication.

Gene A	Chromosome	Length (AA)	Locus position (Mb)	Gene B	Chromosome	Length (AA)	Locus position (Mb)	Score	E-Value	Block	Ka	Ks	Ka/ Ks	Conserved Anchors
GmSHMT06n	Ch06	584	8.46-8.86	GmSHMT04n	Ch04	603	51.90-52.30	28247.0	7e-172	huge	0.02	0.17	0.11	**711**
GmSHMT08c	Ch08	471	8.16-8.56	GmSHMT05c	Ch05	479	34.36-34.76	21948.0	2e-135	huge	0.02	0.20	0.1	551
GmSHMT13ch	Ch13	536	33.32-33.72	GmSHMT15ch	Ch15	244	6.72-7.12	15580	3e-37	large	0.02	0.12	0.16	391
GmSHMT02m	Ch02	514	40.20-40.60	GmSHMT14m	Ch14	507	44.59-44.99	9852.0	2e-158	huge	0.02	0.10	0.16	248
GmSHMT08m	Ch08	518	36.08-36.48	GmSHMT09m	Ch09	517	42.42-42.82	921.0	0.0	large	0.07	0.53	0.13	24
GmSHMT08n	Ch08	566	14.86-15.26	GmSHMT12n	Ch12	563	32.31-32.71	558.0	0.0	large	0.01	0.16	0.06	**14**
GmSHMT08m	Ch08	518	36.08-36.48	GmSHMT02m	Ch02	514	40.20-40.60	361.0	1e-52	Small	0.11	1.64	0.06	**10**
GmSHMT02m	Ch02	514	40.20-40.60	GmSHMT18m	Ch18	518	27.04-27.44	226.0	3e-137	Small	0.10	1.88	0.05	**7**
GmSHMT13m	Ch13	363	18.24-18.44	GmSHMT14m	Ch14	507	32.81-33.01	929.0	0.0	large	0.16	0.29	0.55	**24**

Supplementary Table S2. Number of *SHMT* genes identified in select algae, moss, lycophyte, basal angiosperms, monocotyledonous, and dicotyledonous species available in *Phytozome* (www.phytozome.net). Number of Exons in mitochondrial, chloroplastic, nucleic, and cytosolic targted SHMTs from all analyzed species is shown.

Species	Number of SHMTs	Number of Exons			
		Mitochondrion	Chloroplast	Nucleic	Cytosol
**Green Algae**					
Chlamydomonas reinhardtii	3	16	12	8	
**Moss**					
Physcomitrella patens	14	14	11	4	2
**Lycophyte**					
Selaginella moellendorfii	4	15	10	4	4
**Basal Angiosperm**					
Amborella trichopoda	8	15	7	4	4
**Monocotyledons**					
Hordeum vulgare	3	15	_	4	4
Brachypodium distachyon	4	15	_	4	4
Sorguhum bicolor	4	15	_	4	4
Oryza sativa	5	15	_	4	4
Setaria italica	5	15	_	4	4
Panicum hallii	5	15	_	4	4
Panicum virgatum	8	15	_	4	4
Triticum aestivum	13	15	_	4	4
Zea mays	13	15	_	4	4
**Dicotyledons**					
Carica papaya	5	15	9	4	4
Citrus sinensis	6	15	11	4	4
Cucumis sativus	6	15	11	4	4
Arabidopsis thaliana	7	15	11	4	4 or 3
Solanum lycopersicum	7	15	11	4	4
Capsella rubella	7	15	11	4	4
Fragaria vesca	8	15	11	4	4
Prunus persica	8	15	11	4	4
Populus trichocarpa	9	15	11	4	4
Eucalyptus grandis	10	15	11	4	4
Solanum tuberosum	11	15	11	4	4
Vitis vinifera	11	15	11	4	4
Medicago truncatula	12	15	11	4	4
Glycine max	18	15	11	4	4 or 3

Supplementary Table S3. Primers used for genotyping, expression (qRT-PCR), cloning, subcellular localization, sequencing and EcoTILLING.

Gene	Gene model	Primers	Primer Sequences	Purpose
GmSHMT08c	Glyma08g11490	GmSHMT08-RT-Fw	TAACTTCGCCGTGTTCCCTT	qRT-PCR
		GmSHMT08-RT-Rv	TGTTTCGCGTAGGCCTTAAA	
		GmSHMT08-Fw	ACAACACTCTCTCTTCTCGC	Genotyping
		GmSHMT08-Rv	CAGATTATGAGTTTTGGCCTG	
GmSHMT05c	Glyma05g28490	GmSHMT05-RT-Fw	GTTAATAAGAACGCAGTTT	qRT-PCR
		GmSHMT05-RT-Rv	CAATTCTGATAAGAGAACAGT	
		GmSHMT05-Fw1	GCACACATAGGCACTGGGCAC	Genotyping
		GmSHMT05-Rv1	CTTATCCGCAATTTCCCTGAAGC	
		GmSHMT05-Fw2	AAACGCTTCAGGGAAATTGCGG	
		GmSHMT05-Rv2	GCAAGATCTGTATTCTAGCACATAAAAG	
		GmSHMT05-Fw3	GCGAATTGGTAACCAACTTATTTC	
		GmSHMT05-Rv3	ATAAACCCTACCCCTCCCAGAATGG	
		GmSHMT05-SL-Fw	TATAAAGCTTTTAAAGACGCACACATAGGCAC	Subcellular Localization
		GmSHMT05-SL-Rv	TATAGTCGACTATCCTTGTACTTCATTTCAGA	
GmSHMT06n	Glyma06g11300	GmSHMT06-RT-Fw	GCAGCAGGTGAAGAGAAATGC	qRT-PCR
		GmSHMT06-RT-Rv	TGACACGCCTCACAGACCTTT	
		GmSHMT06-SL-Fw	TATAGAATTCATGGACTCCCAACCGGGTCTC	Subcellular Localization
		GmSHMT06-SL-Rv	TATAGTCGACTAATATCGAATCCTGGCATTGC	
GmSHMT04n	Glyma04g43360	GmSHMT04-RT-Fw	GGCAGCTCAGATTACCAGCAT	qRT-PCR
		GmSHMT04-RT-Rv	ATATCGAAGCCTGGCATTGC	
GmSHMT12n	Glyma12g29171	GmSHMT12-RT-Fw	GTTGTGCCCGCTTTTTACAAA	qRT-PCR
		GmSHMT12-RT-Rv	ATGATGCCACGATCCAAACC	
GmSHMT08n	Glyma08g20050	GmSHMT08b-RT-Fw	TGCAGCTGTCAGGTTTTCACA	qRT-PCR
		GmSHMT08b-RT-Rv	ATGCATTCAGCTGGGATGGT	
		GmSHMT08b-SL-Fw	TATAGAATTCATGGACCTCTCTCACCCTCAA	Subcellular Localization
		GmSHMT08b-SL-Rv	TATAGTCGACTAATGTCAAAACCCGGCATGGC	
GmSHMT14m	Glyma14g36280	GmSHMT14-RT-Fw	GGTTCCTGGAGGCATTCGA	qRT-PCR
		GmSHMT14-RT-Rv	AACTTGACAGCCGCATCAAAG	
GmSHMT02m	Glyma02g38160	GmSHMT02-RT-Fw	GCAAAAAGGAGGTCGAACACTT	qRT-PCR
		GmSHMT02-RT-Rv	TGGTGCGAGTGCTAGCTTTACA	
GmSHMT08m	Glyma08g37270	GmSHMT08c-RT-Fw	GGGAGAGAGCAAAGGAACAAAG	qRT-PCR
		GmSHMT08c-RT-Rv	TTCTTGTGCTTCATGGTTGCTT	
GmSHMT13ch	Glyma13g29410	GmSHMT08c-RT-Fw	CCTAGTTCCAGGAGGCATTCG	qRT-PCR
		GmSHMT08c-RT-Rv	CTTGGAGCTTCGTTCCTGAGA	
GmSHMT18m	Glyma18g27710	GmSHMT18-RT-Fw	GGGAACCCCTGCTCTTACTTCT	qRT-PCR
		GmSHMT18-RT-Rv	TGGCGGAGCTTTGCTATCTC	
GmSHMT09m	Glyma09g33480	GmWEMBL-RT-Fw	GGATTTGCTGAGGAGGATTTTG	qRT-PCR
		GmWEMBL-RT-Rv	GGCGAAGCTTTGCAATCTCT	
Gm-Ubiqutin	Glyma20g27950	GmUBI20-RT-Fw	GTGTAATGTTGGATGTGTTCCC	qRT-PCR
		GmUBI20-RT-Rv	ACACAATTGAGTTCAACACAAACCG	

Supplementary Table S4. The GenBank accessions of the serine hydroxylmethyltransferase (SHMT; EC 2.1.2.1) alleles deposited at NCBI

Line	Amino acid change	GenBank accession
SHMT08_F6266	E61K	KX881924
SHMT08_F6756	M125I	KX881925
SHMT08_F1927	G132D	KX881926
SHMT08_F1817	R257Q	KX881927
SHMT08_F1336	L299F	KX881928
SHMT08_F1801	N368T	KX881929
SHMT08_F891	A302V	KX881930
SHMT08_F427	G71D	KX881931
SHMT08_F1460	G326E	KX881932
SHMT08_Forrest_WT	-	KX881923
SHMT08_Essex_WT	-	KX881933
